# Germinal center entry not selection of B cells is controlled by peptide-MHCII complex density

**DOI:** 10.1038/s41467-018-03382-x

**Published:** 2018-03-02

**Authors:** Chen-Hao Yeh, Takuya Nojima, Masayuki Kuraoka, Garnett Kelsoe

**Affiliations:** 1 Department of Immunology, Duke University School of Medicine, Durham, NC 27710 USA; 2 Duke University Human Vaccine Institute, Duke University School of Medicine, Durham, NC 27710 USA

## Abstract

B cells expressing high affinity antigen receptors are advantaged in germinal centers (GC), perhaps by increased acquisition of antigen for presentation to follicular helper T cells and improved T-cell help. In this model for affinity-dependent selection, the density of peptide/MHCII (pMHCII) complexes on GC B cells is the primary determinant of selection. Here we show in chimeric mice populated by B cells differing only in their capacity to express MHCII (MHCII^+/+^ and MHCII^+/−^) that GC selection is insensitive to halving pMHCII density. Alone, both B cell types generate identical humoral responses; in competition, MHCII^+/+^ B cells are preferentially recruited to early GCs but this advantage does not persist once GCs are established. During GC responses, competing MHCII^+/+^ and MHCII^+/−^ GC B cells comparably accumulate mutations and have indistinguishable rates of affinity maturation. We conclude that B-cell selection by pMHCII density is stringent in the establishment of GCs, but relaxed during GC responses.

## Abstract (web summary)

It has been thought that germinal center (GC) B cells with the highest density of peptide-MHCII molecules receive the most T cell help. Here the authors use heterozygous MHCII inactivation to instead show that after recruitment into the GCs, BCR affinity increases over time independent of the MHC density.

## Introduction

The primary repertoire of B-cell antigen receptors (BCR) is generated by the combinatorial association of V, D, and J gene segments during B-cell development. This primary BCR repertoire is expanded and refined by somatic hypermutation and affinity-driven selection in germinal centers (GC), resulting in a secondary BCR repertoire capable of high affinity binding to virtually any antigen. Selection for entry into nascent GCs seems to be controlled by interclonal competition for T-cell help based on the different levels of peptide/MHC class II (pMHCII) displayed by antigen-activated B cells^1^. Concordantly, even B cells expressing BCRs with very low affinity for antigen can form GCs in the absence of competition from higher-affinity clones^2, 3^. In organized GCs, B cells participate in iterative rounds of interzonal migration, switching between the centroblast state in the GC dark zone (DZ) and the centrocyte state in the light zone (LZ)^4^. Rapid proliferation and fixation of V(D)J mutations characterize the GC DZ, whereas antigen presentation and affinity-dependent selection occur among the T_FH_ and follicular dendritic cells (FDC) in the LZ^5, 6^. Selection in the LZ is thought to represent intraclonal and interclonal competition; the successful B-cell competitors return to the DZ for additional rounds of proliferation and mutation and by this cyclic process maximize the somatic evolution of BCR affinity^7–10^. How FDC and T_FH_ cells function to select higher affinity BCRs from newly mutated B-cell populations, however, is unclear.

Affinity-driven selection in GCs has been proposed to be controlled by the density of pMHCII displayed by B cells during cognate interaction with helper T cells^4^. This “T-cell help” model is supported by mathematical modeling^11, 12^, the finding that BCRs retrieve antigen for processing in an affinity-dependent manner^13^, and the critical function of T_FH_ cells in GC responses^14^. Direct evidence for the role of pMHCII density in controlling GC B-cell competition comes from experiments that deliver antigen to GC B cells by a BCR-independent mechanism that bypasses FDCs^5, 9, 15, 16^. In this experimental model, targeted LZ B cells with increased pMHCII densities have prolonged interaction with T_FH_ cells and preferentially re-enter the DZ for further rounds of proliferation and mutation^5^. These studies also indicate that prolonged, cognate T:B-cell interaction increases the proliferative capacity of GC B cells in the DZ and speeds transit through the cell cycle^9, 15, 16^.

To quantify the role of pMHCII in controlling B-cell selection into and during the GC reaction, we use an alternative strategy to map the limits of T-cell help in the selection of antigen-specific B cells for humoral responses. By short- and long-term B-cell reconstitutions, we place congenic MHCII^+/+^ and haploinsufficient MHCII^+/−^ B cells in direct competition for GC entry and affinity-dependent selection. Even though MHCII expression by B cells is modulated during the course of humoral responses, these competing B-cell populations consistently express twofold differences in MHCII and pMHCII surface density. Our competition experiments confirm that MHCII^+/+^ B cells are preferentially seeded to nascent GCs even though wild type (WT) and haploinsufficient B cells are comparably activated by antigen in vivo. Once GCs are formed, however, MHCII^+/+^ GC B cells have no competitive advantage over haploinsufficient B cells with regard to their persistence, proliferation, acquisition of V(D)J mutations, and affinity maturation. We conclude that pMHCII-driven selection is more stringent for B cells entering GCs than for B cells in established GCs. In this relaxed environment of pMHCII selection, GC B cells with a broad range of BCR affinities can co-exist, increasing the potential for rare evolutionary trajectories to contribute to protective, humoral immunity.

## Results

### MHCII haploinsufficiency does not impair GC responses

**Fig. 1:**
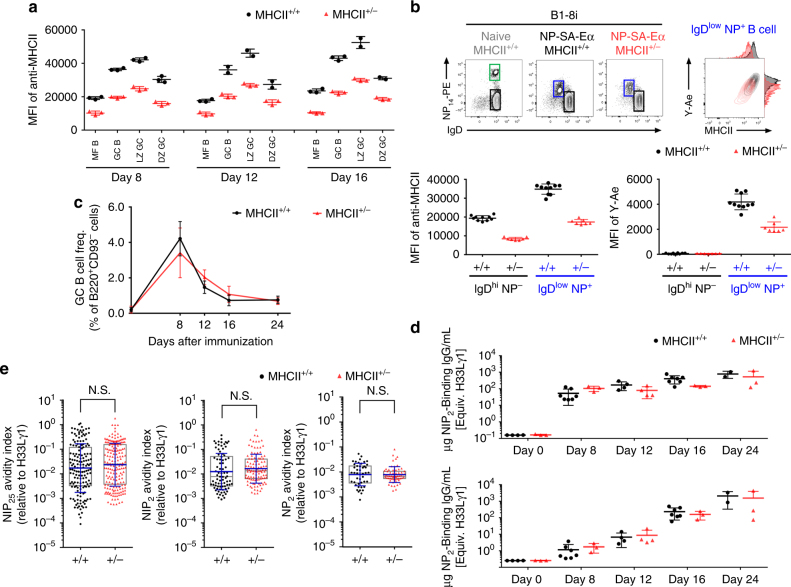
MHCII haploinsufficiency supports normal GC kinetics and affinity maturation. MHCII^+/+^ (black circles) and MHCII^+/−^ (red triangles) mice were immunized i.p. with 20 µg of NP-OVA in alum (**a**, **c**, **d**, **e**). **a** MHCII expression on MF B, GC B, LZ GC B, and DZ GC B cells harvested as indicated post immunization. **b** B1-8.MHCII^+/+^ and B1-8.MHCII^+/−^ mice were immunized in the footpad with 20 µg NP-SA-Eα in alum. Cells from popliteal LNs were analyzed at 16 h. Representative flow plots of IgD expression and NP binding on B220^+^ cells from naïve and immunized B1-8.MHCII^+/+^ mice and immunized B1-8.MHCII^+/−^ mice are shown. Contour plots and histograms representing MHCII expression and Y-Ae-binding (Eα peptide:MHCII complex) on IgD^low^NP^+^B220^+^ cells from B1-8.MHCII^+/+^ (black) and B1-8.MHCII^+/−^ (red) mice. Lower panels show MHCII expression as MFI (left) and Y-Ae-binding (right) in B-cell compartments. **c** Kinetics of GC responses in immunized MHCII^+/+^ and MHCII^+/−^ mice (*n* = 2–5 for both strains at each time point; mean ±S.D.). **d** Kinetics of serum IgG responses (top, NIP_2_-binding IgG; bottom, NP_2_-binding IgG) in MHCII^+/+^ and MHCII^+/−^ mice. IgG concentrations were determined in a Luminex assay in reference to mAb H33Lγ1. Each point represents a single mouse with means (±S.D.) indicated. **e** Single-cell, Nojima cultures for day 8 GC B cells; NIP_25_-, NIP_2_- and NP_2_-specific AvIn values (relative to mAb H33Lγ1) are shown^22^. Each point represents one IgG^+^ clonal culture (*n* = 45–187); boxes represent the 25th, 75th percentiles and median. Bars (blue) indicate the geometric means ±S.D. Statistical significance (*P* < 0.05) was measured by the Mann–Whitney *U* test

Cognate T:B interaction is essential for the initiation and maintenance of GC responses^17, 18^ and the efficacy of these interactions correlates with the quantity of antigen acquired by the B-cell partner^1, 5^. Rather than by introducing targeted antigen^1, 9^, we chose to regulate the availability of pMHCII for cognate T:B interaction by using congenic mice hemizygous for the MHCII locus (MHCII^+/−^)^19^. To quantify the effect of MHCII hemizygosity in various activation states, congenic MHCII^+/+^ and MHCII^+/−^ B6 mice were immunized intraperitoneally (i.p.) with (4-hydroxy-3-nitrophenyl)acetyl (NP)-conjugated ovalbumin (NP-OVA) in alum. Compared with B6 MHCII^+/+^ B cells, MHCII^+/−^ B cells exhibit a haploinsufficiency that reduces MHCII expression by half, on naïve, mature follicular (MF; B220^+^CD93^−^IgD^+^CD138^−^CD38^hi^GL-7^−^) and GC B cells (B220^+^CD38^low^GL7^+^CD95^+^), including GC B cells in the LZ (CXCR4^low^CD86^hi^) and DZ (CXCR4^hi^CD86^low^) compartments (Fig. 1a).

To ensure that MHCII haploinsufficiency equated to a comparable reduction in pMHCIIs, we made a chimeric antigen of NP-streptavidin bound to biotinylated I-Eα52–73 peptide (NP-SA-Eα)^1^. Quantification of Eα52–68 pMHCII complexes can be determined by the Y-Ae monoclonal Ab, which is specific for pEα:I-A^b^ complexes^20^. B1-8.MHCII^+/+^ and B1-8.MHCII^+/−^ mice were immunized in the footpad with NP-SA-Eα in alum; MHCII expression on MF B cells in unimmunized controls was halved in haploinsufficient animals, for both NP-binding and non-binding cells (Supplementary Fig. 1A). In immunized mice, by 16 h post immunization populations of NP-binding B cells with lower membrane IgD levels appeared in both WT and haploinsufficient mice (Fig. 1b). Within these activated IgD^low^NP^+^ B-cell subsets, both MHCII and Eα52–68 pMHCII were halved in MHCII^+/−^ B cells compared to WT controls. This quantitative difference is maintained when B cells are exposed to TLR ligands in vitro (Supplementary Figs. 1B–D).

We observed no significant effects of MHCII haploinsufficiency on T-cell-dependent humoral responses. MHCII^+/+^ and MHCII^+/−^ B6 mice immunized with NP-OVA exhibited comparable IgG Ab levels and GC responses on days 8, 12, 16, and 24 post immunization. Indeed, the kinetics and magnitude of GC responses in MHCII^+/+^ and MHCII^+/−^ mice were indistinguishable (Fig. 1c) and serum IgG for NP and NIP (4-hydroxy-3-iodo-5-nitrophenyl acetyl) were similar as well (Fig. 1d). As expected^21^, heteroclitic (NIP-binding) IgG levels rose faster than NP-specific IgG, but NP- and NIP-specific serum IgG levels converged by day 24 (Fig. 1d). These data demonstrate that MHCII^+/+^ and MHCII^+/−^ B cells have similar intrinsic capacities to produce GCs and serum IgG Ab in response to NP-OVA. In the absence of MHCII^+/+^ competitors, reduced MHCII and pMHCII expression on haploinsufficient B cells does not impact GC responses or affinity maturation of serum IgG Ab.

To determine whether MHCII haploinsufficiency might affect the average or distribution of BCR avidities within GCs, we sorted single MF and GC B cells from the spleens of MHCII^+/+^ and MHCII^+/−^ mice immunized with NP-OVA for single-cell Nojima cultures (Supplementary Fig. 2A)^22^. Eight days post immunization, we obtained a total of 1107 clonal IgG^+^ Nojima cultures. From MHCII^+/+^ mice, we recovered 223 MF and 349 GC IgG^+^ cultures for cloning efficiencies of 73.4% (223/304) and 28.7% (349/1216), respectively. From haploinsufficient, MHCII^+/−^ mice we obtained 227 MF and 308 GC IgG^+^ single-cell cultures with cloning efficiencies of 74.7% (227/304) and 25.3% (308/1216). Similar cloning efficiencies for WT and haploinsufficient MF and GC B cells indicate that MHCII expression levels do not affect MF or GC B-cell survival, proliferation, and plasmacytic differentiation in Nojima cultures.

To compare BCR affinity distributions among GC B cells from MHCII^+/+^ and MHCII^+/−^ mice, we determined the avidity indices (AvIns) for every clonal IgG Nojima culture to NP and NIP^22^. The AvIn represents the ratio of specific (NP- or NIP) binding by individual clonal IgGs to a standard, heteroclitic NP/NIP IgG mAb, H33Lγ1 (*K*_a_ = 2.0 × 10^7^ M^−1^)^21^. We determined AvIn values for both high density (permissive) and low density (stringent), NP- and NIP-binding. The expected, canonical GC response is both heteroclitic (NIP > NP binding) and stringent. From MHCII^+/+^ and MHCII^+/−^ GC, respectively, 44.7% (155/349) and 60.3% (186/308) of clonal IgGs bound to the permissive (low and high avidity), high-density NIP_25_ conjugated Luminex beads; both cohorts exhibited similar AvIn distributions and geometric means that were not significantly different (Fig. 1e). Stringent, heteroclitic binding to NIP_2_ conjugated beads was also identical between the MHCII^+/+^ and MHCII^+/−^ cohorts with similar distributions and geometric means of AvIn values that were comparably higher than those determined for NP_2_ beads (Fig. 1e). In no case did the mean AvIn values for MHCII^+/+^ GC and MHCII^+/−^ GC B cells differ significantly for the same antigen ligand and both WT and haploinsufficient GC B-cell clones exhibited comparable heteroclicity (≅twofold relative to H33Lγ1) for NIP_2_ over NP_2_ (Fig. 1e). We conclude that even at the level of individual GC B-cell clones, MHCII haploinsufficiency has little or no effect on primary GC B-cell responses to NP-OVA.

### MHCII^+/+^ B cells are preferentially recruited into GC responses

**Fig. 2:**
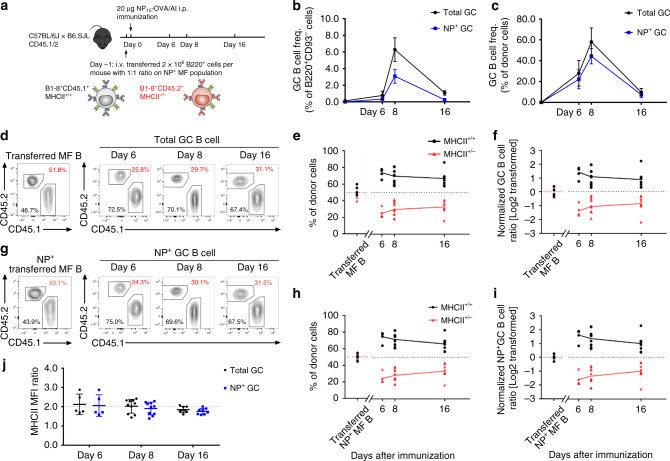
MHCII^+/+^ B cells are advantaged in populating early GCs. **a** Diagrammatic representation of the experimental design. Recipient mice (CD45.1^+^CD45.2^+^) received i.v. transfers of 1:1 mixtures of NP-reactive, congenically marked B1-8.MHCII^+/+^ (CD45.1^+^) and B1-8.MHCII^+/−^ (CD45.2^+^) B cells on day −1. These mice were subsequently immunized i.p. with NP-OVA in alum (day 0). Splenocytes were harvested, labeled and examined by flow cytometry on days 0 (naïve), 6, 8, and 16 post immunization. **b**, **c** Kinetics of **b** total and **c** donor only GC and NP^+^ GC responses. **d**–**f** Proportions of B1-8.MHCII^+/+^ and B1-8.MHCII^+/−^ cells within donor-derived total GC B-cell compartments. **d** Representative flow diagrams, **e** combined results and **f** normalized kinetics from three independent experiments are shown. **g**–**i** Proportions of B1-8.MHCII^+/+^ and B1-8.MHCII^+/−^ cells within donor-derived NP-binding GC B-cell compartments. **g** Representative flow diagrams, **h** combined results, and **i** normalized kinetics from three independent experiments are shown. Numbers indicate the frequencies of cells from B1-8.MHCII^+/+^ (CD45.1^+^, black) and B1-8.MHCII^+/−^ (CD45.2^+^, red) donors (**d**, **g**). Symbols represent the frequencies of CD45.1^+^B1-8.MHCII^+/+^ (black circles) or CD45.2^+^B1-8.MHCII^+/−^ (red triangles) cells among each B-cell compartment of donor cells (**e**, **h**). To normalize against B-cell chimerism variations in individual recipient animals, donor ratios of MHCII^+/+^:MHCII^+/−^ in GC B cells were normalized to the donor ratios of MHCII^+/+^:MHCII^+/−^ in MF B cells (homo/hemi; black). Similarly, donor ratios of MHCII^+/−^:MHCII^+/+^ in GC B cells were normalized to the donor ratios of MHCII^+/−^:MHCII^+/+^ in MF B cells (hemi/homo; red) (**f**, **i**). **j **The ratio of MHCII MFI values on MHCII^+/+^ donors over MHCII^+/−^ donors (total GC B cells, black circles; NP^+^ GC B cells, blue squares). Each symbol represents an individual mouse from at least three independent experiments (*n* = 5–10 at each time point) and the bars indicate the mean values (±S.D.) of each group. The cell populations and gatings were defined as Supplementary Fig. 2

Given their identical, intrinsic capacities for humoral responses, we sought to determine whether MHCII^+/−^ B cells would exhibit lower competitive fitness against MHCII^+/+^ competitors. Consequently, we transferred (2 × 10^6^) MF B cells from congenic CD45.1^+^B1-8^+/+^MHCII^+/+^ and CD45.2^+^B1-8^+/+^MHCII^+/−^ mice into (B6.SJL × B6)F_1_ (CD45.1^+^/CD45.2^+^) recipients (Fig. 2a). The B1-8 VDJ knock-in homogenizes the BCR affinities in both donor cell populations and ensures their activation and competition in response to NP-OVA antigen^23^. Soon after cell transfer (12–16 h), recipient mice were immunized i.p. with NP-OVA in alum. Competition between CD45.1^+^MHCII^+/+^ and CD45.2^+^MHCII^+/−^ splenic B cells was monitored by flow cytometry on days 6, 8, and 16 post immunization (Fig. 2b,c and Supplementary Fig. 2).

Analysis of transferred B cells confirmed a near 1:1 ratio of B1-8^+/+^MHCII^+/+^ and B1-8^+/+^MHCII^+/−^ MF B cells in recipient mice [*n* = 23; CD45.1^+^MHCII^+/+^ 52.8(±5.9)% and CD45.2^+^MHCII^+/−^ 46.1(±5.3)%, respectively] (Fig. 2d). Six days post immunization, low frequencies [0.73(±0.25)%] of GC phenotype B cells (Supplementary Fig. 2) were observed in the spleens of immunized recipients, representing initiation of detectable GC responses (Fig. 2b). Passively transferred, CD45.1^+^MHCII^+/+^ B cells exhibited a significant selective advantage over CD45.2^+^MHCII^+/−^ competitors for acquisition of the GC phenotype; on day 6, the ratio of MHCII^+/+^ [70.9(±5.2)%] to MHCII^+/−^ [28.0(±5.5)%] cells with GC phenotype was approximately 2:1 (Fig. 2d–f). By day 8, the splenic GC compartment expanded almost tenfold, to 6.3(±1.4)% of all mature B cells (B220^+^CD93^−^), but the 2:1 ratio of MHCII^+/+^ [69.7(±8.5)%] to MHCII^+/−^ [29.8(±8.5)%] transferred GC B cells remained essentially constant (Fig. 2d–f). Indeed, this 2:1 ratio was maintained to day 16 [66.9(±9.0)% and 32.7 ± (8.0)%] even as GC responses waned to 1.1(±0.3)% of the mature B-cell compartment. Interestingly, although the fitness advantage of MHCII^+/+^ vs. MHCII^+/−^ GC B cells did not change significantly over time, bias for MHCII^+/+^ B cells at day 6 (2.6:1) fell slightly on days 8 and 16 (2.3:1) (Fig. 2d–f).

To determine whether transferred MHCII^+/+^ and MHCII^+/−^ B cells are comparably activated by immunogen in vivo, we immunized (footpad) transferred mice with NP-SA-Eα in alum. By 16 h post immunization, equivalent populations of IgD^low^NP^+^ B cells were present in both MHCII^+/+^ and MHCII^+/−^ donor cell compartments (Supplementary Fig. 3A) and ratios of activated and resting MHCII^+/+^ and MHCII^+/−^ B cells were identical in immunized and control mice (Supplementary Figs. 3B and 3C). We conclude that even in direct competition, MHCII^+/+^ and MHCII^+/−^ B cells are comparably activated by exposure to antigen. Crucially, MHCII^+/−^ IgD^low^NP^+^ B cells labeled with the Y-Ae mAb were exactly half as bright as WT controls, demonstrating that MHCII and pMHCII are equally reduced in haploinsufficient B cells (Supplementary Figs. 3D and 3E). Lower pMHCII levels on MF B cells do not impair initial activation by antigen.

To ensure that the early fitness advantage of transferred MHCII^+/+^ B cells in the GC response included NP-specific cells, we determined the ratios of NP-binding, CD45.1 MHCII^+/+^ and CD45.2MHCII^+/−^ B cells in the splenic GC compartment (Fig. 2g). This analysis confirmed a nearly 1:1 ratio [50.2(±4.2)% and 49.0(±4.0)%, respectively] of NP-reactive B1-8^+/+^MHCII^+/+^ and B1-8^+/+^MHCII^+/−^ MF B cells were transferred; by day 6 after immunization, however, NP-binding, CD45.1 MHCII^+/+^ B cells constituted 74.7(±6.1)% of donor GC B cells (Fig. 2g–i). Over the course of GC response, the advantage of NP^+^MHCII^+/+^ GC B cells remained relatively constant, ranging from 3:1 on day 6, to 2.5:1 on day 8, and 2:1 on day 16 (Fig. 2g–i). Furthermore, the MHCII expression on CD45.2 MHCII^+/−^ and CD45.1 MHCII^+/+^ GC B and NP^+^ GC cells maintained the twofold difference between haploinsufficient and WT B cells (Fig. 2j). We conclude that the comparable fitness of WT and haploinsufficient B cells in organized GCs includes the antigen-specific B cells and is not an artifact of “dark antigen” responses^22^.

These short-term reconstitution experiments support the notion that pMHCII density controls B-cell entry and/or proliferation in nascent GCs^1^. Nonetheless, whereas MHCII haploinsufficient B cells are strongly disadvantaged in the earliest stages of the GC response, in organized GCs, MHCII^+/+^ and MHCII^+/−^ B cells expressing identical BCR appear to be equally fit.

### No increased fitness for MHCII^+/+^ B cells in organized GCs

**Fig. 3:**
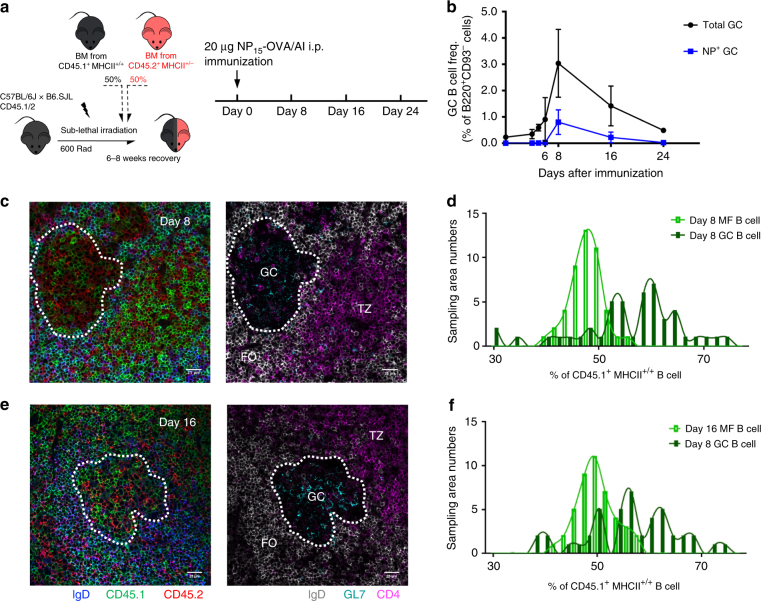
MHCII^+/+^ and MHCII^+/−^ B cells directly compete in GCs. **a** Diagrammatic representation of the experimental design. Sublethally irradiated, recipient mice (CD45.1^+^CD45.2^+^) were reconstituted with a 1:1 ratio of BM cells from MHCII^+/+^ (CD45.1^+^) and MHCII^+/−^ (CD45.2^+^) mice. Six to eight weeks after reconstitution, chimeric mice were immunized i.p. with 20 μg NP-OVA in alum. Splenocytes were harvested, stained and examined by flow cytometry on days 4–24 post immunization. **b** Kinetics of GC B-cell responses are shown (total GC B cells, black circles; NP^+^ GC B cells, blue squares; *n* = 3–13 for both strains at each time point; mean ±S.D.). **c**–**f** Frozen sections from the spleen of day 8 (**c**, **d**) and day 16 (**e**, **f**) chimeras were examined by IHC: IgD (blue and gray), CD45.1 (MHCII^+/+^; green), CD45.2 (MHCII^+/−^; red), GL7 (cyan), and CD4 (magenta). FO follicle, GC germinal center, TZ T-cell zone. Original magnification: ×200, scale bars indicate 25 µm. **d**, **f** Plots represent the distribution of CD45.1^+^MHCII^+/+^ B cells frequency in follicular areas (light green) or GC areas (dark green). Follicular areas (50 × 50 µm; *n* = 44–46 from 22–23 follicles; 2 mice per time point) or GC areas (50 × 50 µm; *n* = 44–46 from 22–23 GCs; *N* = 2 mice per time point) were chosen randomly

To rule out the possibility that the stable persistence of MHCII haploinsufficient GC B cells was due to anatomic isolation of MHCII^+/+^ and MHCII^+/−^ B cells in different GCs, we generated mixed bone marrow (BM) chimeric mice in which half of the reconstituting cells were from CD45.2^+^MHCII^+/−^ hemizygous mice and half from CD45.1^+^MHCII^+/+^WT mice. Equal numbers (5 × 10^6^) of BM cells from both donors were transferred i.v. into sublethally irradiated (B6.SJL × B6)F_1_ (CD45.1^+^/CD45.2^+^) congenic recipients (Fig. 3a). Six- to 8 weeks after transfer, the hematopoietic reconstitution of donor origin cells in the myeloid-, T-, and B-cell compartments were determined by flow cytometry to ensure chimerism in the lymphoid and myeloid compartments; at this time, virtually all B cells are of donor origin (Supplementary Fig. 4). Reconstituted chimeric animals were immunized i.p. with NP-OVA and the magnitude and dynamics of GC responses were found to match those of B6 control mice (Fig. 3b). On day 6 post immunization, chimeric mice exhibit a small but significant [0.9(±0.5)% of B220^+^CD93^−^ cells] population of GC B cells (Fig. 3b). By day 8, chimeric mice had GC B-cell frequencies [3.0(±0.4)%] comparable to those of WT B6 mice (Fig. 1c); the response then waned to 1.6(±0.3)% and 0.5(±0.3)% by days 16 and 24, respectively (Fig. 3b).

Immunofluorescence labeling of histological sections from spleens at 8- and 16 days after immunization show that MHCII^+/+^ and MHCII^+/−^ cells competed in the same GCs (Fig. 3c, e). At both time points, the distributions of CD45.1^+^MHCII^+/+^ B-cell chimerism ranged from 30 to 75% in individual GCs; no GCs populated solely by MHCII^+/−^ or MHCII^+/+^ B cells were observed (Fig. 3d, f). Based on histologic enumeration, GC B-cell populations were significantly biased for CD45.1^+^MHCII^+/+^ B cells (Fig. 3d, f), as observed in short-term transfer experiments (Fig. 2). In contrast, adjacent follicular regions were comparably populated by CD45.2^+^MHCII^+/−^ and CD45.1^+^MHCII^+/+^ B cells (Fig. 3d, f). As expected, MHCII and MHCII-CLIP (class II invariant chain-associated peptide) expression on B cells from both donor genotypes differed twofold in chimeric recipients (Supplementary Fig. 5).

In our short-term transfer studies, diversity in BCR affinity for NP or NIP was minimized by using B1-8 VDJ knock-in donors^23^. To determine whether MHCII haploinsufficient B cells exhibit reduced fitness in GCs when BCR affinity is not constrained, we followed the dynamics of MHCII^+/+^ and MHCII^+/−^ B cells responding to immunization with NP-OVA and asked whether MHCII differences have longer term effects in an environment of direct competition. In the large group of chimeric mice studied (*n* = 56), ratios of MHCII^+/+^ and MHCII^+/−^ MF splenic B cells varied about a mean of ≅2:3 [CD45.1^+^MHCII^+/+^ 37.6(±12.2)% and CD45.2^+^MHCII^+/−^ 60.5(±12.4)%] that matched the ratio of LSK cells in BM. To normalize against variations in B-cell chimerism, ratios of MHCII^+/+^:MHCII^+/−^ GC B cells were normalized to the ratios of MHCII^+/+^:MHCII^+/−^ MF B cells in the same animal.

**Fig. 4:**
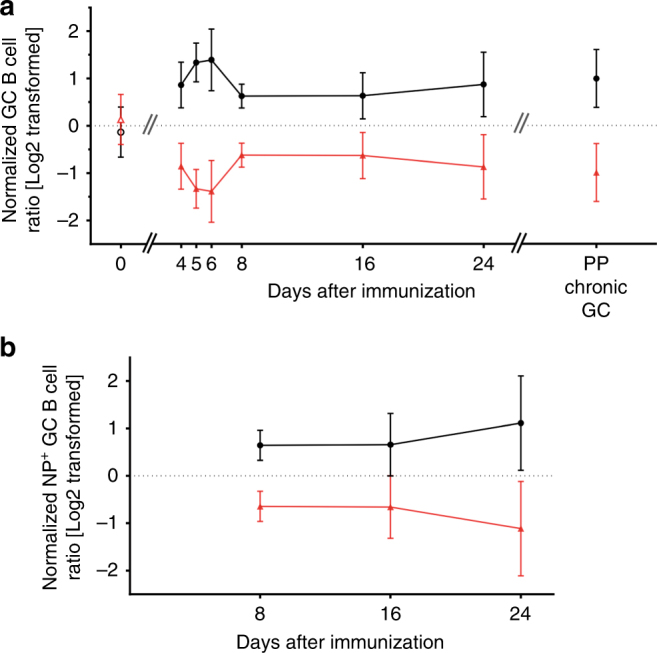
MHCII haploinsufficient GC B cells have robust competitive fitness in GCs. Kinetics of the proportion of MHCII^+/+^ cells and MHCII^+/−^ cells within **a** total GC or **b** NP-binding GC B-cell compartments are shown. To normalize against B-cell chimerism variations in individual recipient animals, donor ratios in day 0 (unimmunized) splenic MF B cells were normalized to the donor ratios in BM LSK cells (MHCII^+/+^/MHCII^+/−^, black hollow circles; MHCII^+/−^/MHCII^+/+^, red hollow triangles). Donor ratios in the GC B cells (days 4–24 spleen and PPs) were normalized to the donor ratios in autologous MF B cells (MHCII^+/+^/MHCII^+/−^, black solid circles; MHCII^+/−^/MHCII^+/+^, red solid triangles). Data combined from three independent experiments (*n* = 3–15 for each time point or group); bars indicate mean values (±S.D.) of each group

As for short term transfers (Fig. 2), CD45.1^+^MHCII^+/+^ B cells in chimeric mice exhibited a significant advantage over CD45.2^+^MHCII^+/−^ B cells for acquisition of the GC phenotype (Fig. 4). By day 4 post immunization, T-cell-dependent B-cell proliferation and acquisition of the GC phenotype^24, 25^ was biased in favor of MHCII^+/+^ B cells (MHCII^+/+^:MHCII^+/−^ = 1.9:1) (Fig. 4a). This bias for MHCII^+/+^ GC-phenotype B cells grew on days 5 and 6, as GCs form and become organized; by day 8, ratios of MHCII^+/+^:MHCII^+/−^ GC B cells stabilized at ≅1.7:1, remaining stable on days 16 and −24 even as GC responses waned to ≅0.5% of the MF B-cell compartment (Fig. 4a). Immunization of BM chimeric mice revealed a common pattern of B-cell competition: B cells with higher MHCII densities are strongly advantaged in the earliest stages of the GC response but exhibit no increase in fitness once the GCs become organized. This ratio (≅2:1) of MHCII^+/+^:MHCII^+/−^ B cells is conserved even in the chronically activated, Peyer’s patch GCs of BM chimeric mice (Fig. 4a).

To determine whether the early competitive advantage of MHCII^+/+^ B cells included the antigen-specific compartment, we determined the ratios of NP-binding, CD45.1^+^MHCII^+/+^ and CD45.2^+^MHCII^+/−^ GC B cells on days 8, 16, and 24 post immunization. Over this period, ratios of NP^+^MHCII^+/+^:NP^+^MHCII^+/−^ GC B cells remained relatively constant, ranging from 1.6:1 on day 8, to 1.7:1 on day 16, and 2:1 on day 24 (Fig. 4b).

### Comparable affinity maturation in MHCII^+/+^ and MHCII^+/−^ GC B cells

It could be that in competition with WT MHCII^+/+^ B cells, haploinsufficient, MHCII^+/−^ GC B cells compensate for diminished pMHCII expression with higher affinity BCRs. Under this model of more stringent selection for MHCII^+/−^ GC B cells, average and distributions of BCR affinities would be generally increased in the haploinsufficient GC B-cell compartment. To test this possibility, we used single-cell Nojima cultures to determine AvIn values for GC B cells from mixed- (CD45.1^+^MHCII^+/+^/CD45.2^+^MHCII^+/−^) and control (CD45.1^+^MHCII^+/+^/CD45.2^+^MHCII^+/+^) chimeric mice. Both groups of BM chimeras were immunized with NP-OVA, and on days 8, 16, and 24 post immunization we sorted single GC B cells for Nojima culture^22^. In this way, we generated 5909 clonal IgG^+^ Nojima cultures with cloning efficiencies ranging from 16 to 21% (Table 1). As before (Fig. 1e), cloning efficiencies and clonal IgG production were comparable between MHCII^+/+^ and MHCII^+/−^ cells.

**Table 1:** ELISA and Luminex summary of single B-cell Nojima cultures

B-cell source^a^	GC (day 8)	GC (day 16)	GC (day 24)
*CD45.1* ^+^ *MHCII* ^+/+^			
IgG^+^/total screened^b^	883/4314	1302/6520	599/3080
Cloning efficiency	20.5%	20.0%	19.5%
NIP_25_^+^ cultures^c^	220 (24.9%)	159 (11.6%)	21 (3.5%)
NIP_2_^+^ cultures^c^	131 (14.8%)	109 (8.4%)	15 (2.5%)
NP_2_^+^ cultures^c^	57 (6.5%)	93 (7.2%)	12 (2.0%)
*CD45.2* ^+^ *MHCII* ^+/+^			
IgG^+^/total screened^b^	705/4314	1141/6,616	579/3080
Cloning efficiency	16.3%	17.2%	18.8%
NIP_25_^+^ cultures^c^	214 (30.3%)	180 (15.8%)	24 (4.1%)
NIP_2_^+^ cultures^c^	152 (21.6%)	151 (13.2%)	18 (3.1%)
NP_2_^+^ cultures^c^	72 (10.2%)	139 (12.2%)	20 (3.5%)

Splenic GC B cells from BM chimera mice were single-sorted into Nojima cultures for in vitro clonal expansion and plasmacytic differentiation on days 8, 16, and 24 after NP-OVA/Alum immunization. Frequencies and of total- and specific IgG in culture supernatants were determined by ELISA and Luminex assays

^a^ GC B cells were sorted from either CD45.1^+^ MHCII^+/+^ or CD45.2^+^ MHCII^+/–^ population at indicated time points

^b^ Number of IgG positive samples/number of samples screened

^c^ Number of Ag-binding IgG positive samples

**Fig. 5:**
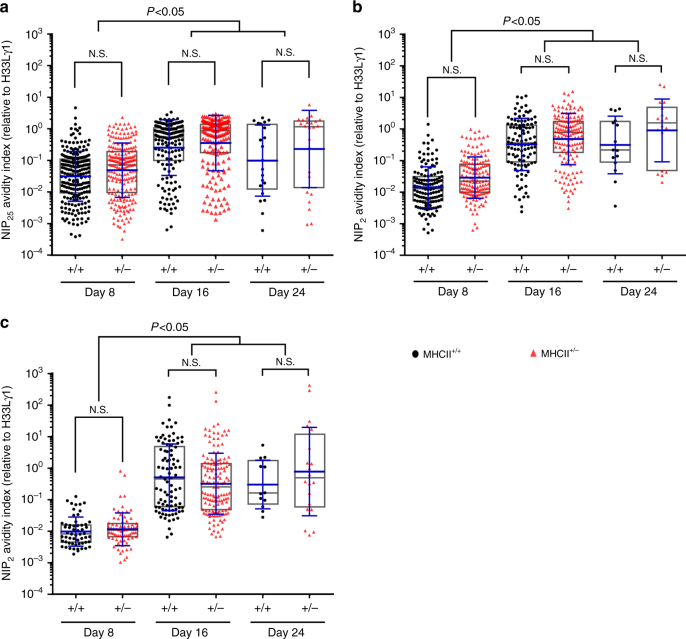
Competing MHCII^+/+^ and MHCII^+/−^ B cells show identical affinity maturation. Single-cell, Nojima cultures were established from splenic GC B cells isolated from BM chimeric mice at days 8, 16, and 24 post immunization (Fig. 3a). **a** NIP_25_-, **b** NIP_2_-, and **c** NP_2_-specific AvIn values (relative to the H33Lγ1 standard) are shown. Each symbol (MHCII^+/+^, black circles; MHCII^+/−^, triangles) represents the AvIn value of one IgG^+^ clonal culture sample (*n* = 12–220, Table 1). Boxes represent the 25th, 75th percentiles and median. Bars (blue) indicate the geometric mean and ±S.D. Statistical significance (*P* < 0.05) was measured using two-way ANOVA with Friedman test followed by Dunn’s multiple comparison post tests. N.S. not significant

AvIns for clonal IgGs from day 8, 16, and 24 GC B cells were determined for high- (permissive) and low density (stringent) NP- and NIP-binding in comparison to the H33Lγ1 standard (Fig. 5 and Supplementary Fig. 6). GC B cell populations from day 8 and 16 exhibited affinity maturation as increased geometric mean AvIn values; mean AvIn for NIP_25_- and NIP_2_-binding increased 8- and 19-fold, respectively, between day 8 and day 16 with no evidence for further affinity maturation at day 24 (Fig. 5 and Supplementary Fig. 6). At each sample time point, AvIn distributions and geometric means of clonal IgGs from MHCII^+/+^ and MHCII^+/−^ GC B cells were comparable. From day 8 GC B-cell cultures, 22.7% (220/883; MHCII^+/+^) and 30.3% (214/705; MHCII^+/−^) of clonal IgGs reacted with NIP_25_-BSA conjugated Luminex beads with both cohorts exhibiting similar AvIn distributions and geometric means (0.03 for MHCII^+/+^ vs. 0.05 for MHCII^+/−^; Fig. 5a and Table 1). As the GC response waned, average AvIn values increased, indicating affinity maturation while fewer clonal IgGs bound NIP_25_ beads [day 16, 11.6% MHCII^+/+^ (159/1302) and 15.8% MHCII^+/−^ (180/1141); day 24, 3.5% MHCII^+/+^ (21/599) and 4.1% MHCII^+/+^ (24/579; MHCII^+/−^)]. Both WT and haploinsufficient cohorts were characterized by similar AvIn geometric means (*P* > 0.05; ANOVA with Friedman test followed by Dunn’s multiple comparison post tests) and distributions (Fig. 5a and Table 1). Stringent binding to NIP_2_- or NP_2_ conjugated beads was also comparable between the MHCII^+/+^ and MHCII^+/−^ GC B-cell competitors with comparable distributions and geometric means of AvIn at each time point (Fig. 5b, c). Heteroclicity, determined by comparing mean AvIn for NIP_2_ vs. NP_2_ beads, was no different between MHCII^+/+^ and MHCII^+/−^ GC B cells (Fig. 5b, c). These patterns of affinity maturation and heteroclicity were virtually identical to that observed in single-cell cultures of CD45.1^+^MHCII^+/+^ and CD45.2^+^MHCII^+/+^ GC B cells from immunized control chimeric mice (Supplementary Fig. 6). Over the course of primary GC responses, BCR affinities of MHCII^+/−^ GC B cells match those of their MHCII^+/+^ competitors.

### Comparable V_H_ mutation in MHCII^+/+^ and MHCII^+/−^ GC B cells

To evaluate the somatic genetics of affinity-driven selection in competing MHCII^+/+^ and MHCII^+/−^ GC B cells, we amplified VDJ rearrangements from 196 IgG^+^ Nojima cultures (102 MHCII^+/+^; 94 MHCII^+/−^) using a multiplex PCR that targets all mouse V_H_ gene segments; amplicons were subsequently cloned, sequenced, and annotated^22^. In 60 VDJ rearrangements from MF B cells (MHCII^+/+^, *n* = 30; MHCII^+/−^, *n* = 30), we observed only a single V_H_ mutation (1 mutation/15,900 bp sequenced; 6.3 × 10^−5^) (Table 2). In contrast, V_H_ mutation frequencies were ≥ 100-fold higher in GC B cells with V_H_ mutation frequencies of 5.6 × 10^−3^ and 4.5 × 10^−3^ for MHCII^+/+^ (*n* = 27) and MHCII^+/−^ (*n* = 25) GC B cells on day 8, and 1.3 × 10^−2^ (MHCII^+/+^, *n* = 44) and 1.5 × 10^−2^ (MHCII^+/−^, *n* = 39) on day 16 (Table 2). Patterns of mutation in both WT and haploinsufficient B cells was also similar in that R:S ratios for MHCII^+/+^ and MHCII^+/−^ GC B cells were comparable: 2.3:1 and 2.0:1  on day 8 and 3.9:1 to 4.0:1 on day 16 (Table 2).

**Table 2:** V_H_ gene sequence summary of single B-cell Nojima cultures

B-cell source^a^		MF (day 8)	GC (day 8)	MF (day 16)	GC (day 16)
*CD45.1* ^+^ *MHCII* ^+/+^					
Total	Clone sequenced^b^	15	27	15	44
	Total base pair sequenced	3981	7161	3972	11,660
	Mutation number	0	40	0	147
	Mutation frequency^c^	N/A	5.59 × 10^−3^	N/A	1.26 × 10^−2^
	R/S ratio^d^	N/A	2.33 (28/12)	N/A	3.90 (117/30)
V_H_1-72^e^	Clone sequenced^b^	0	13	1	11
	Total base pair sequenced	0	3445	265	2915
	Mutation number	0	15	0	45
	Mutation frequency^c^	N/A	4.35 × 10^−3^	N/A	1.54 × 10^−2^
	R/S ratio^d^	N/A	2.00 (10/5)	N/A	5.43 (38/7)
*CD45.2* ^+^ *MHCII* ^+/+^					
Total	Clone sequenced^b^	15	25	15	39
	Total base pair sequenced	3975	6631	3972	10,341
	Mutation number	1	30	0	154
	Mutation frequency^c^	2.51 × 10^−4^	4.52 × 10^−3^	N/A	1.49 × 10^−2^
	R/S ratio^d^	N/A	2.00 (20/10)	N/A	3.97 (123/31)
V_H_1-72^e^	Clone sequenced^b^	0	11	2	15
	Total base pair sequenced	0	2915	530	3975
	Mutation number	0	16	0	69
	Mutation frequency^c^	N/A	5.49 × 10^−3^	N/A	1.73 × 10^−2^
	R/S ratio^d^	N/A	2.20 (11/5)	N/A	6.67 (60/9)

V(D)J rearrangements of cultured B cells were amplified by a nested PCR, cloned, and sequenced. The rearranged V, D, and J gene segments and mutations were identified using IMGT/V-QUEST (http://www.imgt.org/)

^a^ GC B cells were sorted from either CD45.1^+^ MHCII^+/+^ or CD45.2^+^ MHCII^+/**–**^ population at indicated time points

^b^ Number of IgG^+^ Nojima cultures subjected to VDJ sequencing

^c^ Total mutation numbers/total base pair sequenced

^d^ Ratio of replacement/silent mutations

^e^ B cell clones carrying V_H_1-72 rearrangement

These similarities remained when we restricted our analyses to the canonical V_H_1-72 gene segment that dominates the heteroclitic NP responses of B6 mice (Table 2). V_H_1-72 gene segment mutation frequencies and R:S ratios in MHCII^+/+^ and MHCII^+/−^ GC B cells were 4.4 × 10^−3^ and 2.0:1, and 5.5 × 10^−3^ and 2.2:1 on day 8 with comparable increases (1.5 × 10^−2^ and 5.4:1, and 1.7 × 10^−2^ and 6.7:1, respectively) on day 16 (Table 2). Despite their different capacities for MHCII expression, WT and haploinsufficient GC B cells acquire V_H_ mutations in concert and at comparable rates.

## Discussion

BCR affinity maturation in GCs has been proposed to be driven by the quality of T_FH_ and GC B-cell interactions, which in turn are determined by pMHCII density on the B-cell surface^16^. To explore this hypothesis quantitatively, we established by B-cell transfer, a venue for competition between congenic B cells that differed by their capacity to express MHCII. B cells hemizygous for MHCII I-A^b^^19^ were haploinsufficient for MHCII expression^26^ regardless of physiologic state (Figs. 1a, 2j and Supplementary Fig. 5). The haploinsufficiency extended to pMHCII as well; NP-binding B cells from MHCII^+/−^ mice immunized with NP-SA-Eα expressed half the level of Eα peptide/MHCII complex of MHCII^+/+^ controls (Fig. 1b and Supplementary Fig. 3). This quantitative model allows us to explore the role of pMHCII density in clonal selection in humoral immune responses.

MHCII^+/−^ mice mounted serum IgG and GC responses to NP-OVA that were indistinguishable from those of B6 controls (Fig. 1d). This identity shows that pMHCII density per se does not determine the magnitude of humoral responses and recalls the observation that GC responses in B1-8^hi^ and B1-8^lo^ mice exhibit comparable kinetics despite great differences (40-fold) in BCR affinity for NP^23^. However, when MHCII^+/+^ and MHCII^+/−^ B cells directly compete after immunization, MHCII^+/+^ B cells exhibit a significant advantage over MHCII^+/−^ competitors in populating nascent GCs (Fig. 2d–f). This early advantage is not associated with differences in antigen binding, as it persists even when BCR affinities between the competing B cells are homogenized by a shared VDJ knock-in (Fig. 2d–f). This early advantage of MHCII^+/+^ B cells follows B-cell activation, as the appearance and numbers of IgD^low^NP^+^ B cells following immunization with NP immunogens is identical for MHCII^+/+^ and MHCII^+/−^ B cells (Supplementary Fig. 3). We conclude that the early advantage of MHCII^+/+^ B cells reflects pMHCII-dependent selection at the initial, T_H_-dependent entry of antigen-activated B cells into humoral responses^1, 27^.

To our surprise, the early competitive advantage for MHCII^+/+^ B cells was lost once GCs became organized (Figs. 2f and 4a). The skewed ratios of MHCII^+/+^ and MHCII^+/−^ B cells entering GCs become stable and persist without significant change once GC organization is established (Figs. 2f and 4a). Stability in the ratios of MHCII^+/+^:MHCII^+/−^ GC B cells did not reflect anatomical segregation as histologic studies confirmed that all GCs were populated by both MHCII^+/+^ and MHCII^+/−^ B cells (Fig. 3c–f). In GCs, B cells with twofold differences in MHCII expression exhibit the same capacity for persistence within LZ and DZ GC pools. If affinity-dependent competition among GC B cells essentially reflects the “mapping” of BCR affinity onto pMHCII density, our experiments indicate that while MHCII density and T-cell help are limiting factors in pre-GC selection, in established GCs this selection is less stringent. This relaxation of pMHCII selection intensity may support the “permissive” affinity-dependent selection noted for complex protein antigens in established GCs^22^.

Selection on complex phenotypes often results in compensation. For example, mice deficient in CD21/CD35 exhibit reduced serum IgG responses but enhanced affinity maturation^28^ and mixed chimera mice with normal and ICOSL^−/−^ B cells show affinity compensation in ICOSL^−/−^ GC B cells and plasmacytes^29^. To exclude the possibility that MHCII^+/−^ GC B cells compensate for lower pMHCII densities by increased BCR affinity, we determined the BCR AvIns for ≈800 individual MHCII^+/+^ and MHCII^+/−^ GC B cells to NP and NIP (Fig. 5). Heteroclitic, affinity maturation was highly similar between MHCII^+/+^ and MHCII^+/−^ GC B cells, with no compensatory affinity increases observed in MHCII^+/−^ GC B cells (Fig. 5 and Supplementary Fig. 6). Mean AvIn values did not differ significantly between MHCII^+/+^ and MHCII^+/−^ GC B cells at any sample time and AvIn distributions were comparable in each cohort (Fig. 5). Direct measurement of BCR avidity obviates the limitations of predicting affinity maturation by enumerating affinity-enhancing mutations^29^.

Immunofluorescence studies of immunized chimeric mice showed that all GCs comprised B cells from both MHCII^+/+^ and MHCII^+/−^ donors, demonstrating that these B cells competed in common GC niches (Fig. 3c–h). That both groups exhibited similar AvIn distributions, including comparable lower avidity “tail” populations (Fig. 5)^22, 30^, ensures that spatial segregation cannot account for persistence and continuing affinity maturation of MHCII^+/−^ GC B cells.

We find it highly unlikely that pMHCII on MHCII^+/+^ and MHCII^+/−^ GC B cells are ever equalized. Bannard et al. have reported that MHCII molecules turn over rapidly in DZ B cells by ubiquitin-mediated degradation^31^, perhaps to ensure that pMHCII density accurately represents BCR affinity. If so, pMHCII densities on WT and haploinsufficient B cells would reflect the abundance of the immediate precursor, the MHCII-CLIP complex, which is halved also in MHCII^+/−^ GC B cells (Supplementary Fig. 5).

The notion that GC B cells with higher pMHCII densities are advantaged in GCs by enhanced T_FH_ help came from experiments in which antigen linked to DEC-205 mAb (αDEC-205-Ag) was delivered to DEC-205^+^ GC B cells^5, 9^. This targeted loading of antigen is BCR and FDC independent but correlated with increased B:T_FH_ interaction, DZ proliferation, and V_H_ mutation frequencies^9, 15^. We see no evidence for these effects when competing B cells acquire antigen via their BCR. What is surprising is that B-cell entry into GC responses is strongly affected by a twofold reduction in MHCII and pMHCII (Figs. 2 and 4). If the GC represents the paradigm for affinity-dependent, B-cell selection, it seems counter-intuitive that T:B interactions that initiate humoral responses are more stringent than those within the GC.

Results from other experimental models that establish competition between B cells with higher- or lower levels of MHCII expression are similar to our own. For example, in the absence of H2-O expression, B cells with elevated pMHCII densities exhibit a pronounced advantage over WT B cells on entry into GCs, but once GCs become established ratios of H2-O-deficient and -sufficient GC B cells remain constant for 21 days^32^. Likewise, Bannard et al. infected mixed BM chimeric mice containing B cells that expressed normal MHCII or MHCII resistant to ubiquitination mediated turnover with influenza^31^. One week after infection, ubiquitin resistant GC B cells represented 10−50% of GC B cells while at 5 weeks these cells remained almost as abundant at 0–40%^31^. Finally, immunization of mixed BM chimeric mice containing WT and CD83^−/−^ B cells (with lower MHCII expression) with SRBC resulted in an early preference for CD83-sufficient GC B cells, but with little or no change in CD83^+/+^:CD83^−/−^ ratios between days 6 and 12 post immunization^33^.

The permissive nature of selection in GCs^22^ was recently underscored when Turner et al. showed that B cells responding to a dissimilar antigen could enter into and persist within an ongoing GC response^34, 35^. These authors show that HyHEL-10 B cells specific for hen- (HEL) and duck egg lysozyme (DEL) exposed ex vivo to DEL-OVA enter and proliferate in GCs elicited by DEL-OVA or OVA immunization with equal efficiency^35^. One surprising interpretation of this result is that the B cell’s initial exposure to antigen determines its fitness for the GC response rather than the ability to recover antigen repeatedly from GC FDCs. If these results^34, 35^ are generalizable, current models for affinity-dependent selection in GCs require substantial revision. At least one possibility is that in the LZ, low affinity GC B cells have a substantial chance of receiving “bystander help” from local T_FH_ activated by fitter cells. Such unspecific help would be consistent with recent results from the Nussenzweig laboratory^36^ showing that apoptosis in the GC LZ is essentially independent of BCR affinity.

The GC is a dynamic microenvironment where antigen-activated B cells iteratively undergo proliferation, hypermutation, and affinity-driven selection. By direct observation, selection for higher BCR affinities in GCs is rapid and relies on the unequal success of mutant B cells in generating progeny. Nonetheless, GC B-cell populations also comprise substantial subsets of mutated B cells with very low BCR avidities and GC selection may be functionally permissive for these less fit populations^22, 30^. Given that pathogens evolve under selection by host immunity, permissive selection may be a strategy to optimize memory B-cell compartments against pathogen variants that have escaped immune control. The development of broadly neutralizing antibodies to influenza and HIV from B-cell clonal lineages characterized by extraordinary frequencies of V(D)J mutation are consistent with this tortuous pathway to protective efficacy^37–40^.

In summary, by allowing congenic MHCII^+/+^ and MHCII^+/−^ B cells to compete directly, we show that WT and haploinsufficient GC B cells exhibit similar fitness in GCs as determined by their proliferation, persistence, mutation frequencies, and affinity maturation. In contrast, haploinsufficient B cells are significantly disadvantaged during the initiation of humoral responses, most likely during the initial T:B collaboration that marks the start of humoral responses. If affinity-driven selection is determined by pMHCII density on B cells, that mechanism appears to be significantly more stringent at the initiation of the humoral response than during the GC reaction itself.

## Methods

### Mice and immunizations

C57BL/6, B6.SJL-Ptprc^a^ Pepc^b^/BoyJ (CD45.1^+/+^), B6.129S2-H2^dlAb1-Ea^/J (MHCII^−/−^), and B6.129P2 (C)-^Ightm2Cgn^/J (B1-8i^+/+^) female mice were purchased from the Jackson Laboratory and were maintained under specific pathogen-free, temperature- and humidity-controlled conditions at the Duke University Animal Care Facility and used in experiments at 8–12 weeks of age. Due to the limited availability of knockout and chimeric mice, no randomization was used. The investigator was not blinded to the group allocation during the animal experiments. Sample size to ensure adequate statistical power was based on prior experience in the laboratory. Mice were immunized i.p. with 20 μg of NP_15_-OVA/Alhydrogel adjuvant 2% (1:1, v/v) (Biosearch Technologies and InvivoGen) in a final volume of 200 µL. Serum and spleen samples were collect on days 4–24 after immunization. All experiment procedures involving animals were approved by the Duke University Institutional Animal Care and Use Committee.

### Short-term transfer and mixed bone marrow chimeric mice

For short-term cell transfers, single-cell suspensions were harvested and processed from spleens of B1-8^+/+^MHCII^+/+^ (CD45.1^+^) and B1-8^+/+^MHCII^+/−^ (CD45.2^+^) or B1-8^+/+^MHCII^+/+^ (CD45.2^+^) mice. Splenocytes were stained with biotinylated-Abs (αCD4, αCD11c, αCD43, αCD90.2, αF4/80, and αGL-1) and subsequently labeled with Streptavidin MicroBeads (Miltenyi Biotec). B cells were then negatively purified using magnetic activated cell sorting with CS column on a VarioMACS separator (Miltenyi Biotec). After sorting, purified B cell samples were stained and examined using flow cytometry to determine the purity and percentage of NP^+^ populations. 100 μL of cell mixtures containing 2 × 10^6^ cells with 1:1 ratios of NP^+^CD45.1^+/+^B1-8^+/+^MHCII^+/+^ and NP^+^ CD45.2^+/+^B1-8^+/+^MHCII^+/−^ B cells were transferred i.v. to individual recipient (B6.SJL × B6) F_1_ (CD45.1^+^/CD45.2^+^) mice. To generate mixed BM chimeric mice, (B6.SJL × B6) F_1_ (CD45.1^+^/CD45.2^+^) mice were sub-lethally irradiated with a single dose of 6.5 Gy X-ray and then injected i.v. with equal numbers (2.5 × 10^6^ cells) of BM cells harvested from B6.SJL (CD45.1^+^) and MHCII^+/−^ (CD45.2^+^) or B6 (CD45.2^+^) mice. Reconstituted mice were rested for 6–8 weeks before use in experiments.

### Antibodies and flow cytometry

For surface marker detection, samples were resuspended in PBS containing 0.5% bovine serum albumin, 0.1% sodium azide and 1 mM EDTA (FACS buffer). Resuspended cells were blocked with rat anti-mouse CD16/32 (2.4G2) and rat IgG (Sigma-Aldrich) in FACS buffer for 30 min and stained with fluorochrome-conjugated antibodies specific for CD19 (1D3), CD21/35 (7E9), CD23 (B3B4), CD38 (90), CD43 (S7), CD45.1 (A20), CD45.2 (104), CD86 (GL-1), CD93 (AA4.1), CD95 (Fas; Jo2), CD138 (281-2), CD184 (CXCR4; L276F12), B220 (RA3-6B2), GL-7 (GL7), I-A/I-E (M5/114.15.2), I-Ab (AF6-120.1), IgD (11-26 c.2a), IgM (II/41) and MHC-Clip (15G4) or NP_14_-PE chemical (Biosearch Technologies) at 4 °C for 30–40 min (Supplementary Table 1). LIVE/DEAD Fixable Near-IR Dead Cell Stain Kit (Life Technologies) was used to determine the viability of cells. Subsequently, cells were washed twice with FACS buffer, and the resuspended cells were then analyzed on LSRII or LSRFortessa cell analyzer (BD Biosciences). Fluorescence activated cell sorting was acquired on MoFLo Astrios sorter (Beckman Coulter), FACSVantage SE sorter with DIVA Option or FACSAria sorter (BD Biosciences). The data analysis was performed using FACSDiva (BD Biosciences) and FlowJo software (Tree Star). Specific cell population and gating strategy are described in Supplementary Fig. 2.

### Single B-cell culture

Single MF and GC B cells from control mice and immunized mice were expanded in the presence of NB-21.2D9 feeder cells (Nojima cultures) as described^22^. Briefly, NB-21.2D9 feeder cells (2000 cells/well) were pre-seeded into 96 well culture plate with RPMI 1640 media (Life Technologies) supplemented with 10% FBS (Thermo Fisher Scientific), 10 mM HEPES, 1 mM sodium pyruvate, 1× MEM nonessential amino acid, 100 U/mL penicillin–streptomycin and 5.5 × 10^−5^ M 2-mercaptoethanol (All Life Technologies). Next day, single MF and GC B cells were directly sorted into NB-21.2D9 culture plates and cultured in the presence of exogenous recombinant IL-4 (2 ng/ml, Peprotech) and CD154/BAFF/IL-21-expressing NB-21.2D9 cells. Two-thirds of culture media were replaced with fresh media daily from days 2 to −8. On day 10, culture supernatants and cell pellets were harvested for subsequent ELISA IgG determinations and V(D)J amplifications, respectively.

### ELISA and luminex multiplex immunoassays

Concentrations of IgG in harvested culture supernatants were determined by standard sandwich ELISA as described^22^. Briefly, 384-well ELISA plates (Corning) were coated with anti-mouse Igκ Ab and anti-mouse Igλ Ab (2 μg/ml each; Southern Biotech) in carbonate buffer overnight at 4 °C. After blocking with PBS containing 0.5% BSA for 1 h at room temperature, serially diluted samples (1:100, 1:1000, and 1:2000 dilutions) or the H33Lγ1 mAb standard were applied to the plate and incubated overnight at 4 °C. After washing, HRP-conjugated anti-mouse IgG (1:5000 dilution; Southern Biotech) was added and incubated at room temperature for 1 h. After washing, bound HRP activity was visualized using a TMB peroxidase kit (BioLegend) and the optical densities (O.D.) were determined at 450 nm with a SpectraMax M2 Microplate Reader (Molecular Devices). IgG^+^ supernatants screened by ELISA and serum samples were subjected to Luminex Multiplex Immunoassay for the detection of avidity against different antigen as reported^41^. Briefly, 1 × 10^7^ coded MicroPlex microspheres (Luminex) were covalently linked to 50 µg of NP_2_-BSA, NP_30_-BSA, NIP_2_-BSA, NIP_25_-BSA (Biosearch Technologies), OVA (Sigma-Aldrich), Igκ, Igλ, or IgG (Southern Biotech), respectively. Conjugated microsphere mixture were incubated with diluted samples for 2 h at room temperature. After washing, any IgG Ab bound to the microspheres was detected with 2 µg/ml PE goat anti-mouse IgG (Southern Biotech). Fluorescence was measured on a Bio-Plex 3D suspension array system (Bio-Rad Laboratories).

### Immunohistochemistry

Harvested spleens were embedded in Tissue-Tek OCT Compound (Sakura Finetek) and frozen at −80 °C. 5–10 µm-thick sections were mounted on glass slides and rehydrated by soaking in wash solution (PBS containing 0.5% BSA and 0.1% Tween 20) at RT for 30 min. Samples were then blocked with rat anti-mouse CD16/CD32 (90) and rat IgG (Sigma-Aldrich) for 15 min at room temperature. After washing, the samples were incubated with CD4 (GK1.5), CD45.1 (A20), CD45.2 (104), GL-7 (GL7), and IgD (11-26c.2a) in a humid, dark chamber for 3 h at RT or 4 °C overnight (Supplementary Table 1). After washing, the samples were then incubated with secondary or enhancing antibodies for 1 h at RT. Images were acquired by confocal microscopy using a Zeiss LSM 780 confocal microscope (Zeiss; ×200 magnification). CD45.1 and CD45.2 positive B cells in GC or B-cell follicle area were quantified using ImageJ software (National Institutes of Health).

### BCR repertoire analysis and Ig SHM

V(D)J rearrangements of cultured B cells were amplified by a nested PCR as described^22^. Briefly, total RNA was extracted from selected culture cell pellets using TRIzol or TRIzol LS reagents (Invitrogen) and cDNA was subsequently synthesized using Superscript III with oligo (dT)_20_ primers. The cDNA were then subjected to two rounds of PCR with established primers^42, 43^. V(D)J amplicons were gel purified, ligated into vectors, and transformed into bacteria for further sequencing. DNA sequencing were performed at Duke DNA sequencing facility. The rearranged V, D, and J gene segments and mutation numbers were identified using IMGT/V-QUEST (http://www.imgt.org/). The replacement/silent mutation (R/S) ratio were calculated based on the sequenced V_H_ exon region (FR1 to the start of CDR3).

### Statistical analysis

Statistical comparisons were determined using two-tailed Student’s *t* test with Mann–Whitney’s *U* test or analysis of variance (ANOVA) with Kruskal–Wallis test (one-way) or Friedman test (two-way) followed by Dunn’s multiple comparison post tests. Differences were considered statistically significant when the *P* values < 0.05.

### Data availability

Sequence data that support the findings of this study have been deposited in GenBank with the accession codes MF942137-MF942331.

## Electronic supplementary material

Supplementary Information
