# Supplementary material for: Germinal center entry not selection of B cells is controlled by peptide-MHCII complex density
Source: Nat Commun. 2018 Mar 2;9:928. doi: 10.1038/s41467-018-03382-x (PMC5834622; doi:10.1038/s41467-018-03382-x)
Supplement: Supplementary file 1 — Supplementary Information [file 41467_2018_3382_MOESM1_ESM.docx]

SUPPLEMENTAL MATERIALS

The limits of MHC Class II-driven selection in germinal centers

**Authors:** Chen-HaoYeh^1^, Takuya Nojima^1^, Masayuki Kuraoka^1^, and Garnett Kelsoe^1, 2^*

**Affiliations:**

^1^Department of Immunology, Duke University School of Medicine, Durham, NC 27710, USA.

^2^Duke University Human Vaccine Institute, Duke University School of Medicine, Durham, NC 27710, USA.

*Corresponding author

**Correspondence:** Garnett Kelsoe

Department of Immunology, Duke University

Durham, North Carolina 27710, USA

Tel: (919) 613-7815; Fax: (919) 684-8982; E-mail: ghkelsoe@duke.edu

**
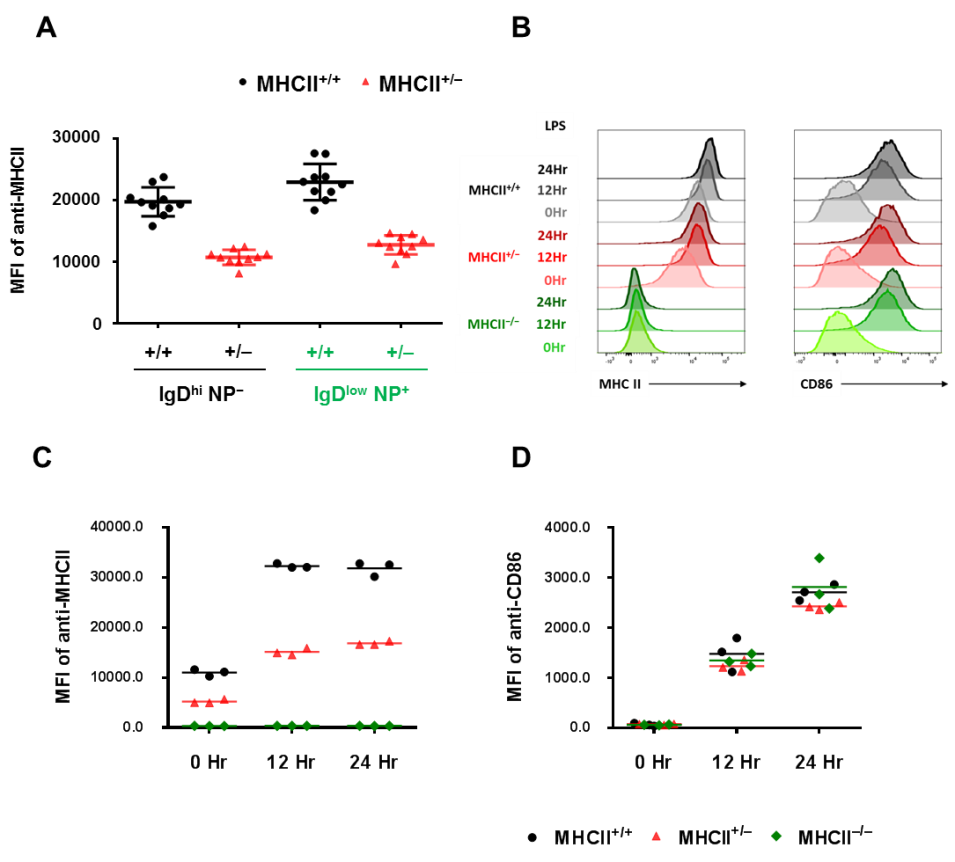
**

**Supplementary Figure 1. Haploinsufficiency of MHCII on naïve B cells did not impair their responsiveness to TLR ligand activation *in vitro*. (A)** Haploinsufficiency of MHCII on IgD^hi^NP^+^ and IgD^hi^NP^−^ naïve B cells from individual B1-8.MHCII^+/+^ and B1-8.MHCII^+/−^ mice. B220^+^ B cells from the pLN of naïve B1-8.MHCII^+/+^ and B1-8.MHCII^+/−^ mice were harvested, stained and examined by flow cytometry. Plot represents the MFI of MHCII expression on IgD^hi^NP**^−^** (black) or IgD^hi^NP**^+^** (green) populations as defined in Fig. 1B. Each symbol represents an individual mouse from two independent experiments and the bars indicate the mean values (± SD) of each group (n=10). **(B-D)** Splenic B220^+^ B cells from MHCII^+/+^, MHCII^+/−^ and MHCII^−/−^ mice were activated with 5 μg/mL LPS (lipopolysaccharide) for 0, 12 and 24 hours *in vitro*. Representative histograms (**B**) and combined results (**C** and **D**) of the MHCII and CD86 expression on LPS-stimulated B cells. (**C** and **D**) Plots represent the MFI of MHCII and CD86 expression**.** Symbols represent the samples from MHCII^+/+^ (black circles), MHCII^+/−^ (red triangles) or MHCII^−/−^ (green diamonds) mice and the bars indicate the mean of each group.

**
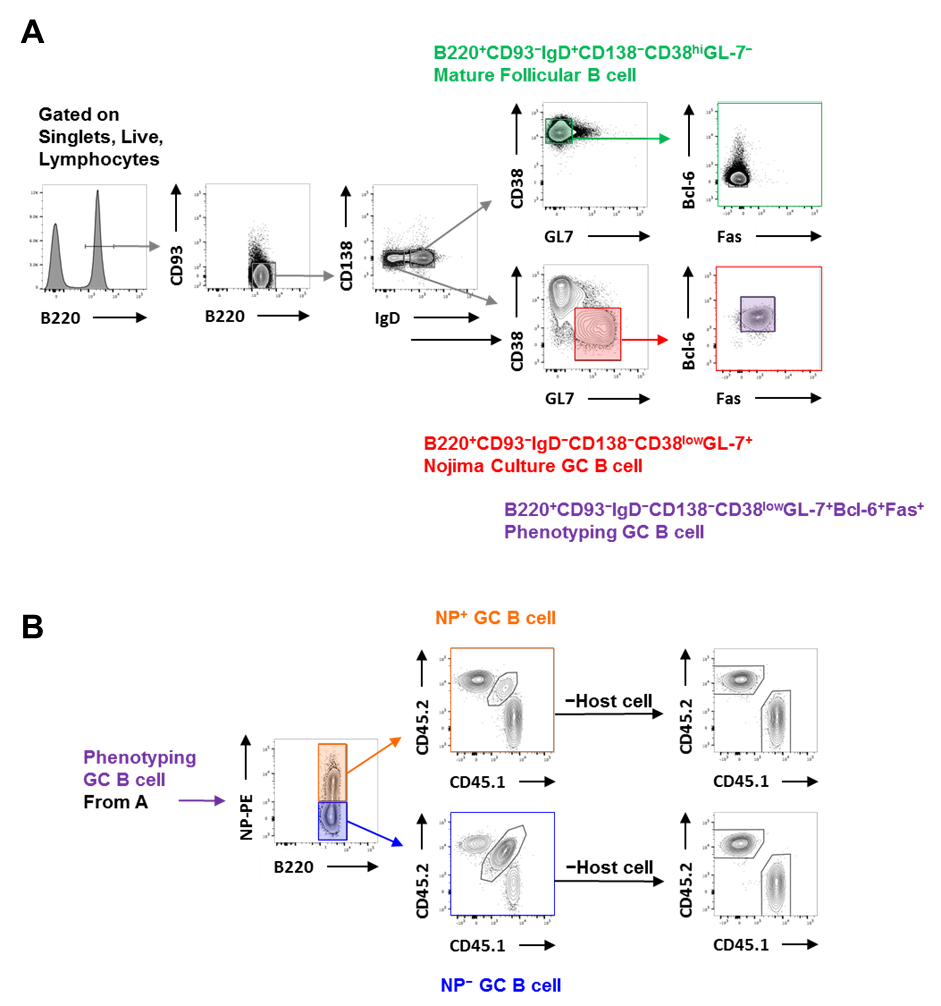
**

**Supplementary Figure 2. Gating strategy to isolate MF B cells and GC B cells.** Splenocytes harvested from immunized mice were pre-gated on single-lymphocytes using the height, width and area of forward and side scattered light. Dead cells were excluded using the LIVE/DEAD™ reagents. (**A**) For single-cell Nojima culture, MF B (green) and GC B (red) cells were defined as B220^+^CD93^−^IgD^+^CD138^−^CD38^hi^GL-7^−^ and B220^+^CD93^−^IgD^−^CD138^−^CD38^low^GL-7^+^ populations, respectively. For phenotypic analysis, GC B cells (purple) were defined as B220^+^CD93^−^IgD^−^CD138^−^CD38^low^GL-7^+^Bcl-6^+^Fas^+^ population. (**B**) For short-term adoptive transfers and long-term BM chimeras, GC B cells (Fig. S2A; purple) were further divided by expression of NP-PE, CD45.1 and CD45.2 for the analysis of antigen-specific, donor-derived GC B cells.


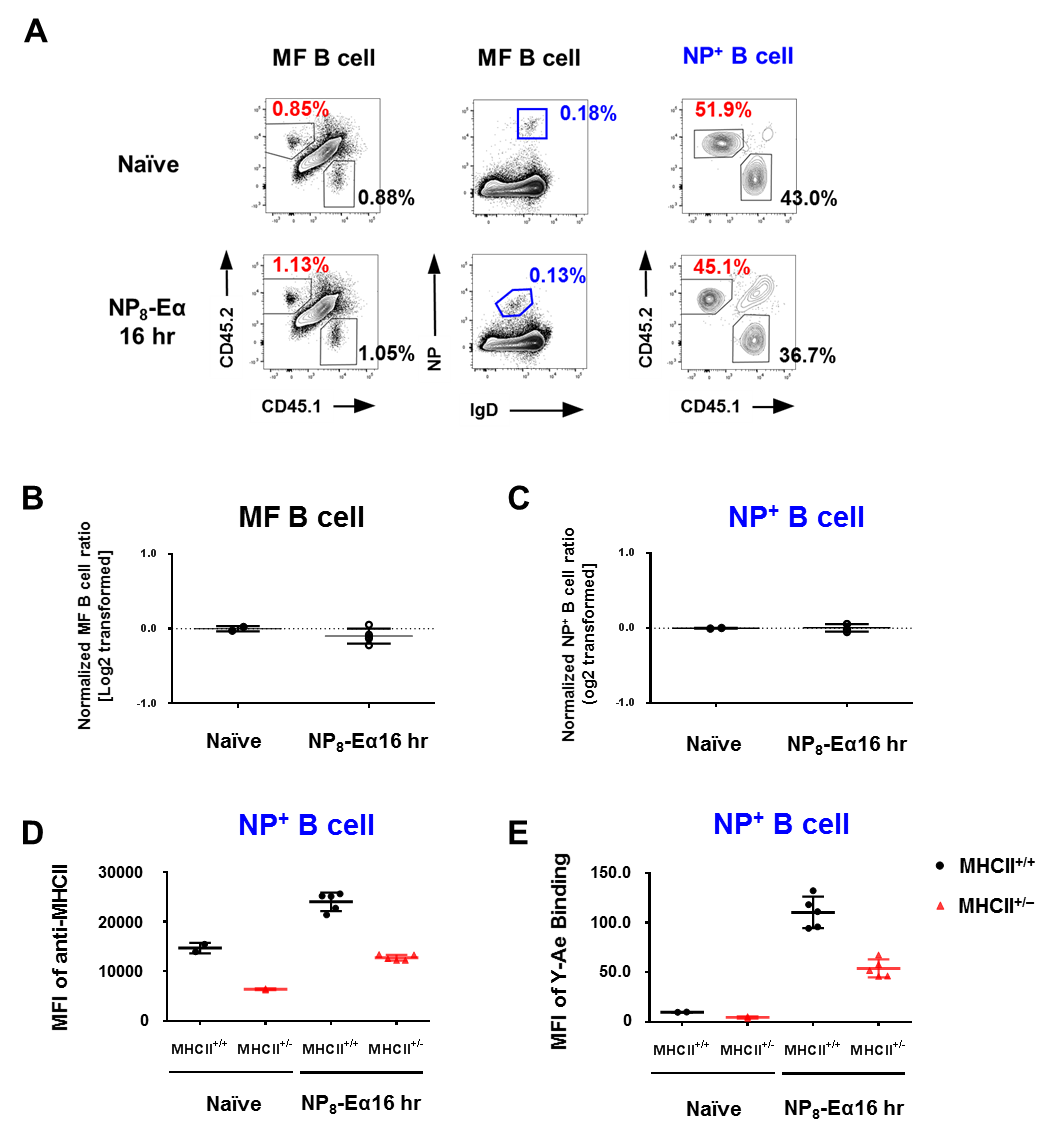


**Supplementary Figure 3. Adoptively transferred MHCII^+/+^ and MHCII^+/−^ MF B cells are equally activated after immunization.** Recipient mice received 1:1 ratio of NP^+^ B1-8.MHCII^+/+^ (CD45.1^+^) and B1-8.MHCII^+/−^ (CD45.2^+^) MF B cells (Fig. 2A) were then immunized with 20 µg of NP-SA-Eα in alum via footpad. Splenocytes were harvested, stained and examined by flow cytometry on 16 hrs post-immunization. (**A**) Frequency of transferred B1-8.MHCII^+/+^ and B1-8.MHCII^+/−^ cells within total- and NP-binding MF B-cell compartments (NP^hi^IgD^hi^ in naïve mice and NP^low^IgD^low^ in immunized mice). Numbers indicate the frequencies of B1-8^+/+^MHCII^+/+^ (black) and B1-8^+/+^MHCII^+/−^ (red) derived cells in each gated population. (**B** and **C**) Dot plots represent the normalized MHCII^+/+^:MHCII^+/−^ donor ratio in total MF (**B**) and NP-binding MF (**C**) B-cell compartments as defined in Fig. S2A. The cell populations and normalization equation were defined as Fig. 2. (**D** and **E**) Dot plots represent the MFI of MHCII-expression (**D**) and Y-Ae binding (**E**) on NP^+^ B cells. Data were from one experiment (n = 7).

**
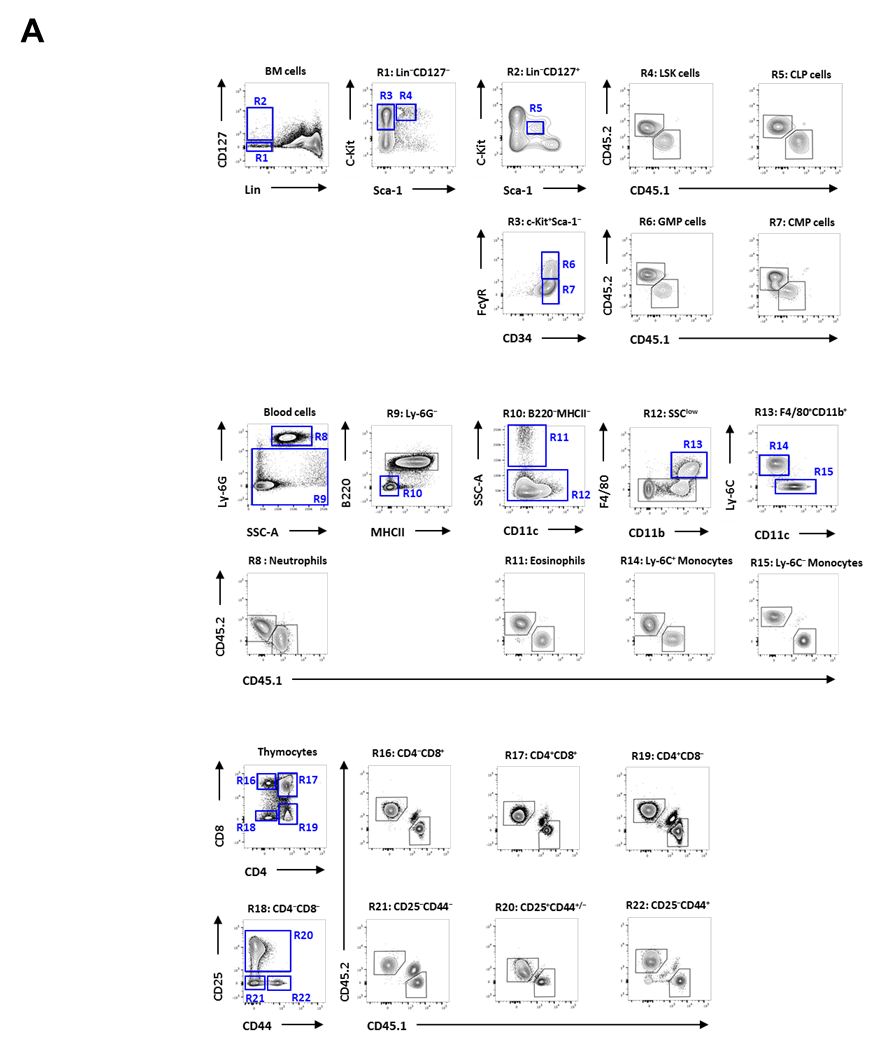

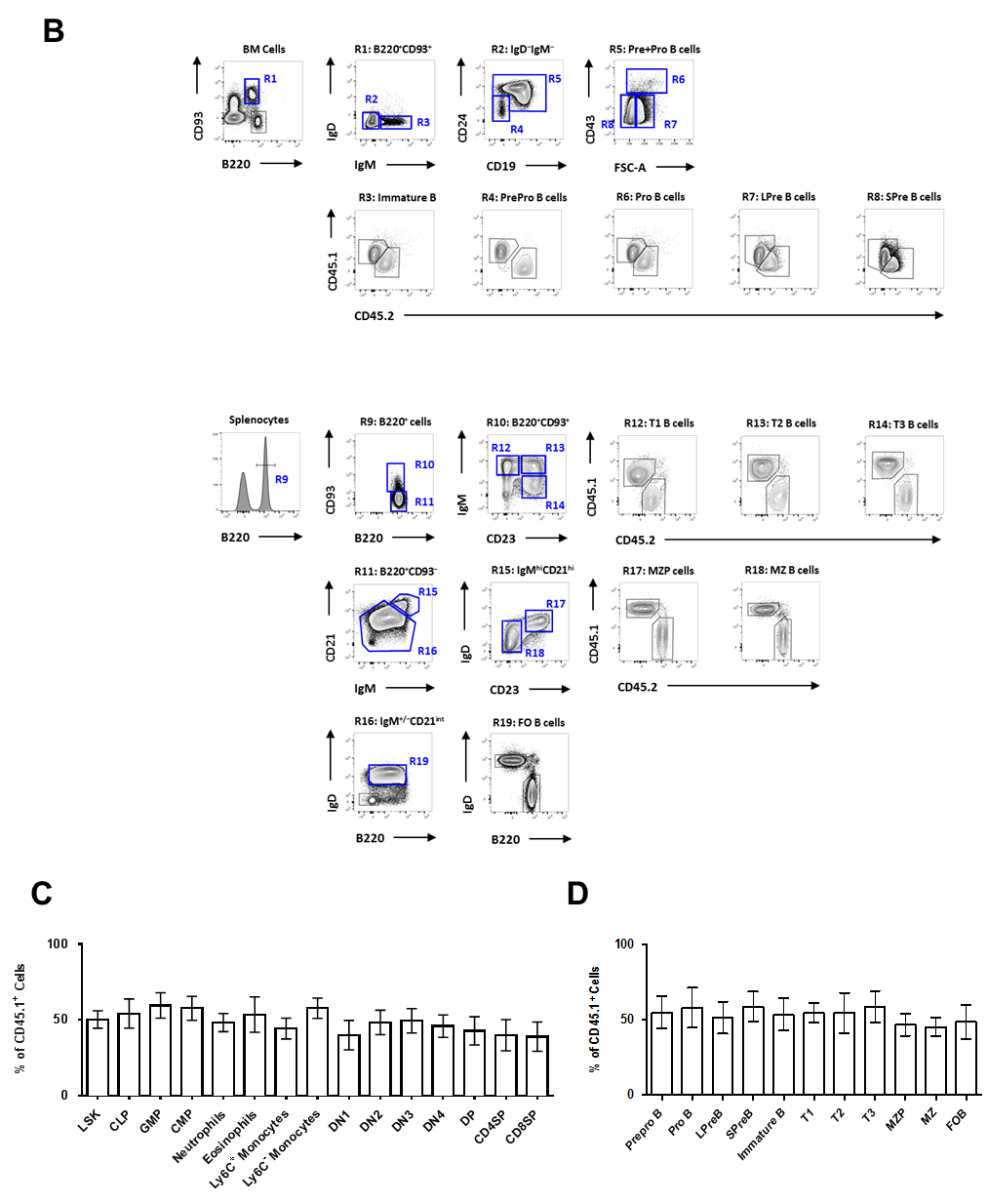
**

**Supplementary Figure 4. Identification of donor-derived CD45.1^+^CD45.2^−^ cells and CD45.1^−^CD45.2^+^ cells in progenitors, myeloid- and T-lineage cell compartments of mixed BM chimeric mice. (A)** CD127^−^Lin^−^ (CD4^−^CD8^−^CD11b^−^CD11c^−^CD19^−^B220^−^Gr-1^−^Ter119^−^) BM cells (R1) were divided by Sca-1, c-Kit, CD34, and FcγR expression into LSK (R4), granulocyte macrophage progenitor (GMP; R6) and common myeloid progenitor (CMP; R7) compartments. CD127^+^Lin^−^ (CD4^−^CD8^−^CD11b^−^CD11c^−^CD19^−^B220^−^Gr-1^−^Ter119^−^) BM cells (R2) were divided by Sca-1 and c-Kit, expression into common lymphoid progenitor (CLP; R5) compartment. Blood cells were divided by Ly-6G expression into Ly-6G^+^ Neutrophil compartment (R8) and Ly-6G^−^ compartment (R9). Ly-6G^−^B220^−^MHCII^−^ blood cells (R10) were divided by SSC-A, CD11c, CD11b, F4/80 and Ly-6C expression into eosinophil (R11), Ly-6C^+^ monocytes (R14) and Ly-6C^−^ monocytes (R15) compartments. Thymocytes were divided by expression pattern of CD4 and CD8 into CD8 single positive (R16), double positive (R17), double negative (R18) and CD4 single positive (R19) compartments. Double negative compartment (R18) were further divided by CD25 and CD44 expression into CD25^−^CD44^−^ (DN1; R21), CD25^+^ (DN2/3; R20) and CD25^−^CD44^+^ (DN4; R22) compartments. **(B)** Developing B-cell compartment (R1) in the BM was identified by B220 and CD93 expression, and was further divided by IgM and IgD expression into Pro/Pre (R2) and immature/T1 (R3) B-cell compartments. B220^+^CD93^+^IgM^−^IgD^−^ BM cells (R2) were further divided by CD19, CD24, CD43 and FSC-A expression into CD19^−^CD24^−^ Prepro B-cell (R4), CD19^+^CD43^+^ Pro B-cell (R6), CD19^+^CD43^−^FSC-A^hi^ large Pre B-cell (R7) and CD19^+^CD43^−^FSC-A^low^ small Pre B-cell (R8) compartments. Splenic developing (R10) and mature (R11) B-cell compartments were identified by B220 and CD93 expression. B220^+^CD93^−^ immature B-cell (R10) was further divided by IgM and CD23 expression into IgM^+^CD23^−^ T1 B-cell (R12), IgM^+^CD23^+^ T2 B-cell (R13) and IgM^−^CD23^+^ T3 B-cell (R14) compartments. Mature B-cell compartment (R11) was further divided by IgM, IgD, CD21 and CD23 expression into IgM^hi^CD21^hi^CD23^+^ marginal zone progenitor (MZP; R17), IgM^hi^CD21^hi^CD23^−^ marginal zone B-cell (MZ; R18) and IgM^int/-^IgD^+^CD21^int^CD23^+^ follicular B-cell (FO; R19) compartments. **(C)** Summary charts represent the frequency of CD45.1^+^ (MHCII^+/+^) population in total donor-derived cells (CD45.1^+^ or CD45.2^+^) as gated in Fig S4A. **(D)** Summary charts represent the frequency of CD45.1^+^ (MHCII^+/+^) population in total donor-derived cells (CD45.1^+^ or CD45.2^+^) as gated in Fig. S4B. The bars indicate the mean values (± SD) of each cell population from one experiment (n = 8 chimeric mice).

**
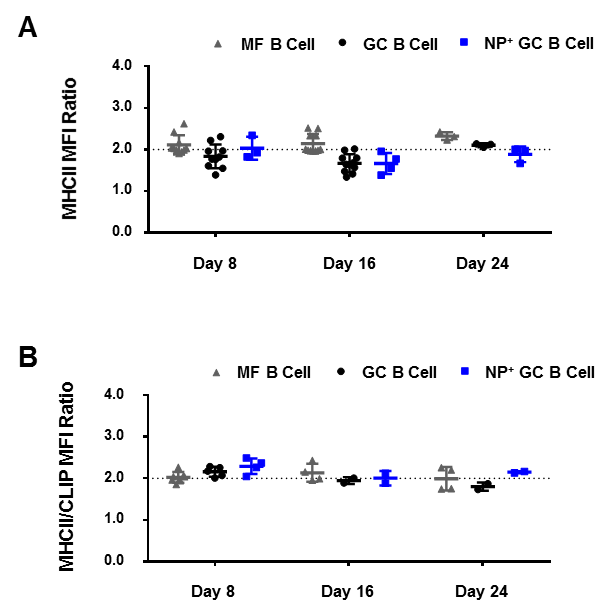
**

**Supplementary Figure 5. Haploinsufficiency of donor cells in the mixed BM chimera experiments.** Splenocytes from mixed BM chimeric mice (Fig. 3A) were harvested, labeled and examined by flow cytometry on days 8-24 after *i.p.* immunization with NP-OVA in alum. Dot plot represent the ratio of MFI for (**A**) MHCII and (**B**) MHCII/CLIP complex (MHCII^+/+^ cells over MHCII^+/−^ cells) expression on MF B cells (gray triangles), GC B cells (black circles) and NP^+^ GC B cells (blue squares). Each symbol represents an individual mouse and the bars indicate the mean values (± S.D.) of each group. **A;** Data were pooled form 3 independent experiments (n=24). **B;** Data were form 1 experiment (n=8).


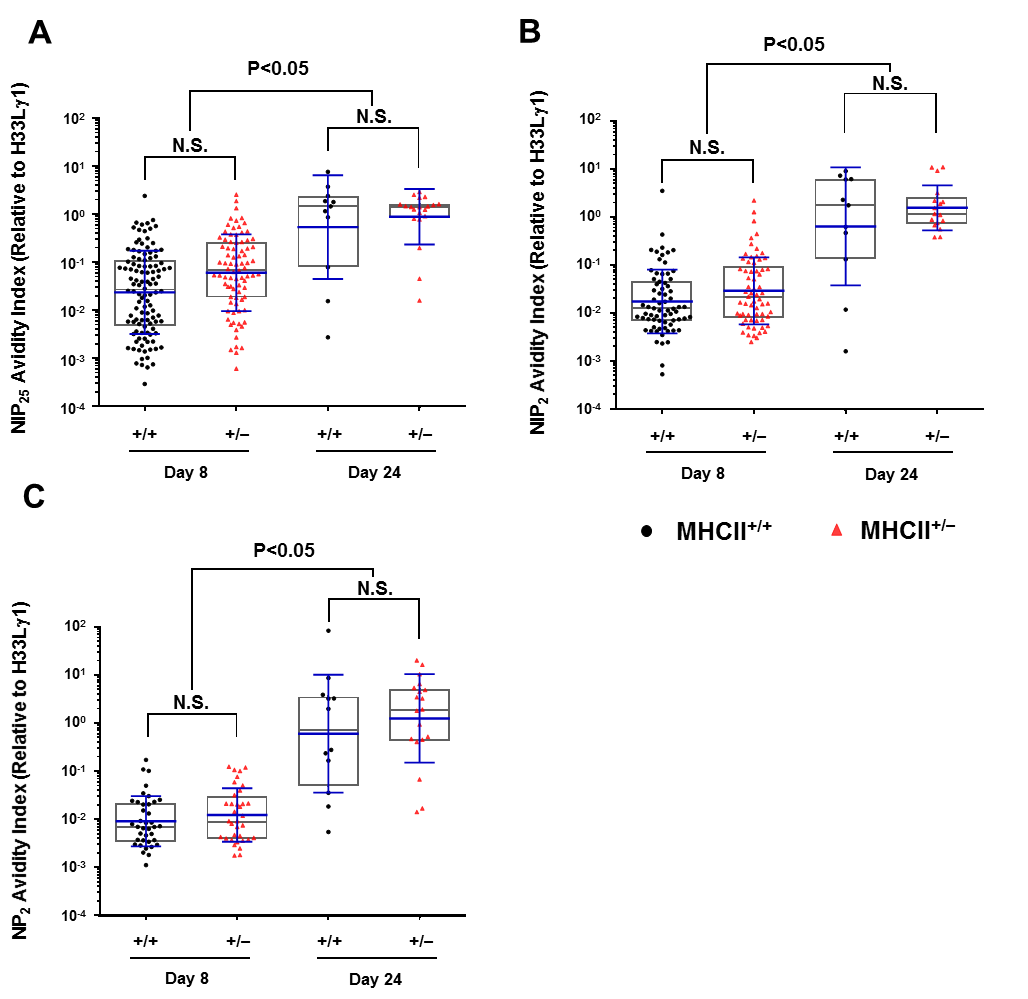


**Supplementary Figure 6. Comparable affinity maturation of CD45/Ly-5 congenic GC B cells in control BM chimeric mice.** Splenocytes from control mixed BM chimeric mice (Fig. 3A; 50% CD45.1^+^MHCII^+/+^ and 50% CD45.2^+^MHCII^+/+^) were harvested, labeled and examined by flow cytometry on days 8 and 24 after immunization with NP-OVA in alum. Single B cells from splenic GC B cells were sorted into Nojima cultures. Plots represent the distributions of NIP_25_- (**A**), NIP_2_- (**B**) and NP_2_-specific (**C**) AvIn values relative to standard H33Lγ1 antibody. Each symbol indicates an AvIn value for an individual clonal IgG^+^ culture sample (CD45.1^+^MHCII^+/+^, black circles; CD45.2^+^MHCII^+/+^, red triangles; n =12-112). Box plots (gray) illustrate the 25% percentile, median and 75% percentile. Horizontal bars (blue) indicate the geometric mean values with geometric SD of each group. Statistical significance (P < 0.05) was determined using two-way ANOVA with Friedman test followed by Dunn’s multiple comparison post-tests. N.S., not significant.

**Supplementary Table 1. Antibodies and staining reagents used in this study.**

| **Number** | **Specificity** | **Clone** | **Reactivity** | **Isotype** | **Fluorochrome** | **Con. mg/mL** | **Vender** | **Titration** |
| --- | --- | --- | --- | --- | --- | --- | --- | --- |
|  |  |  |  |  |  |  |  |  |
| **1** | **CD3** | **145-2C11** | **Mouse** | **Hamster IgG1** | **BV711** | **0.2** | **BioLegend** | **1/200** |
| **2** | **CD4** | **GK1.5** | **Mouse** | **Rat IgG2a, κ** | **Biotin** | **0.5** | **BioLegend** | **1/500** |
| **3** | **CD4** | **GK1.5** | **Mouse** | **Rat IgG2a, κ** | **BV510** | **0.2** | **BioLegend** | **1/200** |
| **4** | **CD8a** | **53-6.7** | **Mouse** | **Rat IgG2a, κ** | **Biotin** | **0.5** | **BioLegend** | **1/500** |
| **5** | **CD8a** | **53-6.7** | **Mouse** | **Rat IgG2a, κ** | **PE-Cy7** | **0.2** | **BioLegend** | **1/200** |
| **6** | **CD11b** | **M1/70** | **Mouse** | **Rat IgG2b, κ** | **Biotin** | **0.5** | **BioLegend** | **1/500** |
| **7** | **CD11b** | **M1/70** | **Mouse** | **Rat IgG2b, κ** | **PE-Cy7** | **0.2** | **BioLegend** | **1/100** |
| **8** | **CD11c** | **N418** | **Mouse** | **Hamster IgG** | **APC** | **0.2** | **BioLegend** | **1/100** |
| **9** | **CD19** | **6D5** | **Mouse** | **Rat IgG2a, κ** | **AF488** | **0.5** | **BioLegend** | **1/500** |
| **10** | **CD19** | **1D3** | **Mouse** | **Rat IgG2a, κ** | **BV786** | **0.2** | **BD** | **1/200** |
| **11** | **CD21/CD35** | **7E9** | **Mouse** | **Rat IgG2a, κ** | **Biotin** | **0.5** | **BioLegend** | **1/500** |
| **12** | **CD21/35** | **7G6** | **Mouse** | **Rat IgG2b, κ** | **FITC** | **0.5** | **BioLegend** | **1/200** |
| **13** | **CD21/35** | **7E9** | **Mouse** | **Rat IgG2a, κ** | **PE-Cy7** | **0.2** | **BioLegend** | **1/500** |
| **14** | **CD23** | **B3B4** | **Mouse** | **Rat IgG2a, κ** | **Biotin** | **0.5** | **BioLegend** | **1/200** |
| **15** | **CD23** | **B3B4** | **Mouse** | **Rat IgG2a, κ** | **PerCP e710** | **0.2** | **eBioscience** | **1/200** |
| **16** | **CD23** | **B3B4** | **Mouse** | **Rat IgG2a, κ** | **AF647** | **0.2** | **BioLegend** | **1/200** |
| **17** | **CD24** | **M1/69** | **Mouse** | **Rat IgG2b, κ** | **Biotin** | **0.5** | **BioLegend** | **1/200** |
| **18** | **CD24** | **M1/69** | **Mouse** | **Rat IgG2b, κ** | **AF488** | **0.5** | **BioLegend** | **1/200** |
| **19** | **CD25** | **PC61** | **Mouse** | **Rat IgG1, λ** | **BV421** | **0.2** | **BioLegend** | **1/200** |
| **20** | **CD34** | **RAM34** | **Mouse** | **Rat IgG2a, κ** | **FITC** | **0.5** | **eBioscience** | **1/200** |
| **21** | **CD38** | **90** | **Mouse** | **Rat IgG2a, κ** | **PerCP e710** | **0.2** | **eBioscience** | **1/200** |
| **22** | **CD38** | **90** | **Mouse** | **Rat IgG2a, κ** | **PE-Cy7** | **0.2** | **BioLegend** | **1/200** |
| **23** | **CD43** | **S7** | **M/H** | **Rat IgG2a, κ** | **Biotin** | **0.5** | **BD** | **1/500** |
| **24** | **CD43** | **S7** | **M/H** | **Rat IgG2a, κ** | **APC** | **0.2** | **BD** | **1/200** |
| **25** | **CD44** | **IM7** | **M/H** | **Rat IgG2b, κ** | **FITC** | **0.5** | **BioLegend** | **1/200** |
| **26** | **CD45.1** | **A20** | **Mouse** | **Mouse (A.SW) IgG2a, κ** | **PE** | **0.2** | **BioLegend** | **1/200** |
| **27** | **CD45.1** | **A20** | **Mouse** | **Mouse (A.SW) IgG2a, κ** | **PE/594** | **0.2** | **BioLegend** | **1/200** |
| **28** | **CD45.1** | **A20** | **Mouse** | **Mouse (A.SW) IgG2a, κ** | **AF647** | **0.2** | **BioLegend** | **1/200** |
| **29** | **CD45.1** | **A20** | **Mouse** | **Mouse (A.SW) IgG2a, κ** | **BV421** | **0.2** | **BioLegend** | **1/200** |
| **30** | **CD45.1** | **A20** | **Mouse** | **Mouse (A.SW) IgG2a, κ** | **BV650** | **0.2** | **BD** | **1/200** |
| **31** | **CD45.2** | **104** | **Mouse** | **Mouse (SJL) IgG2a, κ** | **BUV395** | **0.2** | **BD** | **1/200** |
| **32** | **CD45.2** | **104** | **Mouse** | **Mouse (SJL) IgG2a, κ** | **FITC** | **0.5** | **eBioscience** | **1/200** |
| **33** | **CD45.2** | **104** | **Mouse** | **Mouse (SJL) IgG2a, κ** | **PE** | **0.2** | **BioLegend** | **1/200** |
| **34** | **CD45.2** | **104** | **Mouse** | **Mouse (SJL) IgG2a, κ** | **PE-Cy7** | **0.2** | **BioLegend** | **1/200** |
| **35** | **CD86** | **GL-1** | **Mouse** | **Rat IgG2a, κ** | **PE** | **0.2** | **BioLegend** | **1/200** |
| **36** | **CD86** | **GL-1** | **Mouse** | **Rat IgG2a, κ** | **PE-Cy7** | **0.2** | **eBioscience** | **1/200** |
| **37** | **CD93** | **AA4.1** | **Mouse** | **Rat IgG2b, κ** | **Biotin** | **0.5** | **eBioscience** | **1/400** |
| **38** | **CD93** | **AA4.1** | **Mouse** | **Rat IgG2b, κ** | **APC** | **0.2** | **BioLegend** | **1/200** |
| **39** | **CD93** | **AA4.1** | **Mouse** | **Rat IgG2b, κ** | **BV421** | **0.2** | **BD** | **1/200** |
| **40** | **CD95 (Fas)** | **Jo2** | **Mouse** | **Hamster IgG2, λ2** | **Biotin** | **0.5** | **BD** | **1/200** |
| **41** | **CD95 (Fas)** | **Jo2** | **Mouse** | **Hamster IgG2, λ2** | **PE/594** | **0.5** | **BD** | **1/200** |
| **42** | **CD117 (c-Kit)** | **2B8** | **Mouse** | **Rat IgG2b, κ** | **APC** | **0.2** | **BD** | **1/100** |
| **43** | **CD127** | **SB/199** | **Mouse** | **Rat IgG2b, κ** | **PE** | **0.2** | **BD** | **1/100** |
| **44** | **CD138** | **281-2** | **Mouse** | **Rat IgG2a, κ** | **Biotin** | **0.2** | **BD** | **1/400** |
| **45** | **CD138** | **281-2** | **Mouse** | **Rat IgG2a, κ** | **PE** | **0.2** | **BioLegend** | **1/400** |
| **46** | **CD138** | **281-2** | **Mouse** | **Rat IgG2a, κ** | **BV421** | **0.2** | **BD** | **1/400** |
| **47** | **CD138** | **281-2** | **Mouse** | **Rat IgG2a, κ** | **BV605** | **0.2** | **BD** | **1/400** |
| **48** | **B220** | **RA3-6B2** | **M/H** | **Rat IgG2a, κ** | **AF700** | **0.5** | **BioLegend** | **1/200** |
| **49** | **B220** | **RA3-6B2** | **M/H** | **Rat IgG2a, κ** | **BV421** | **0.2** | **BioLegend** | **1/200** |
| **50** | **B220** | **RA3-6B2** | **M/H** | **Rat IgG2a, κ** | **BV605** | **0.2** | **BD** | **1/200** |
| **51** | **B220** | **RA3-6B2** | **M/H** | **Rat IgG2a, κ** | **BV786** | **0.2** | **BioLegend** | **1/200** |
| **52** | **Bcl-6** | **K112-91** | **M/H** | **Mouse IgG1, κ** | **PE** | **50 test** | **BD** | **1/100** |
| **53** | **Bcl-6** | **K112-91** | **M/H** | **Mouse IgG1, κ** | **PE/594** | **50 test** | **BD** | **1/100** |
| **54** | **Ea52-68/I-Ab** | **eBioY-Ae** | **Mouse** | **Rat IgG2b, κ** | **Biotin** | **0.2** | **eBioscience** | **1/200** |
| **55** | **Ea52-68/I-Ab** | **eBioY-Ae** | **Mouse** | **Rat IgG2b, κ** | **FITC** | **0.2** | **eBioscience** | **1/200** |
| **56** | **F4/80** | **BM8** | **Mouse** | **Rat IgG2a, κ** | **Biotin** | **0.5** | **BioLegend** | **1/500** |
| **57** | **GL7** | **GL7** | **M/H** | **Rat IgM, κ** | **FITC** | **0.5** | **BD** | **1/200** |
| **58** | **GL7** | **GL7** | **M/H** | **Rat IgM, κ** | **PerCP e710** | **0.2** | **eBioscienceio** | **1/200** |
| **59** | **GL7** | **GL7** | **M/H** | **Rat IgM, κ** | **AF647** | **0.2** | **BD** | **1/200** |
| **60** | **Gr1** | **RB6-8C5** | **Mouse** | **Rat IgG2b, κ** | **Biotin** | **0.5** | **BioLegend** | **1/500** |
| **61** | **I-A / I-E** | **M5/114.15.2** | **Mouse** | **Rat IgG2b, κ** | **AF647** | **0.5** | **BioLegend** | **1/400** |
| **62** | **I-A / I-E** | **M5/114.15.2** | **Mouse** | **Rat IgG2b, κ** | **BV711** | **0.2** | **BD** | **1/400** |
| **63** | **I-Ab** | **AF6-120.1** | **Mouse** | **BALB/c IgG2a, κ** | **PerCP e710** | **0.2** | **eBioscience** | **1/400** |
| **64** | **I-Ab** | **AF6-120.1** | **Mouse** | **BALB/c IgG2a, κ** | **PE/594** | **0.2** | **BD** | **1/400** |
| **65** | **IgD** | **11-26c.2a** | **Mouse** | **Rat IgG2a, κ** | **Biotin** | **0.5** | **BD** | **1/200** |
| **66** | **IgD** | **11-26c.2a** | **Mouse** | **Rat IgG2a, κ** | **BV510** | **0.2** | **BD** | **1/200** |
| **67** | **IgM** | **II/41** | **Mouse** | **Rat IgG2a, κ** | **FITC** | **0.2** | **eBioscience** | **1/200** |
| **68** | **IgM** | **II/41** | **Mouse** | **Rat IgG2a, κ** | **PerCP e710** | **0.2** | **eBioscience** | **1/200** |
| **69** | **IgM** | **II/41** | **Mouse** | **Rat IgG2a, κ** | **PE-Cy5** | **0.2** | **eBioscience** | **1/200** |
| **70** | **Ig Lambda** | **187.1** | **Mouse** | **Rat IgG2a, κ** | **FITC** | **0.2** | **BD** | **1/200** |
| **71** | **Ig Lambda** | **RML42** | **Mouse** | **Rat IgG2a, κ** | **APC** | **0.2** | **BioLegend** | **1/200** |
| **72** | **Ly-6C** | **AL21** | **M/H** | **Rat IgM, κ** | **FITC** | **0.5** | **BD** | **1/100** |
| **73** | **Ly-6G** | **1A8** | **M/H** | **Rat IgG2a, κ** | **PE** | **0.2** | **BD** | **1/100** |
| **74** | **MHCII-CliP** | **15G4** | **Mouse** | **Mouse IgG1, κ** | **FITC** | **100 test** | **SantaCruz** | **1/20** |
| **75** | **TCRβ** | **H57-597** | **Mouse** | **Hamster IgG2, λ1** | **BV711** | **0.2** | **BD** | **1/200** |
| **76** | **TER-119** | **TER-119** | **Mouse** | **Rat IgG2b, κ** | **Biotin** | **0.5** | **BioLegend** | **1/500** |
| **77** | **NP_14_-PE** | **--** | **--** | **--** | **PE** | **1** | **LGC Biosearch** | **1/200** |
| **78** | **Streptavidin** | **--** | **--** | **--** | **BB515** | **0.1** | **BD** | **1/200** |
| **79** | **Streptavidin** | **--** | **--** | **--** | **PE** | **0.2** | **BioLegend** | **1/400** |
| **80** | **Streptavidin** | **--** | **--** | **--** | **PE-Cy5** | **0.2** | **eBioscience** | **1/400** |
| **81** | **Streptavidin** | **--** | **--** | **--** | **PE-Cy7** | **0.2** | **BioLegend** | **1/200** |
| **82** | **Streptavidin** | **--** | **--** | **--** | **APC** | **0.2** | **BioLegend** | **1/200** |
| **83** | **Streptavidin** | **--** | **--** | **--** | **BV421** | **0.1** | **BD** | **1/200** |
| **84** | **Propidium Iodide** | **--** | **--** | **--** | **PI** | **1** | **LifeTech** | **1/5000** |
| **85** | **L/D Near IR** | **--** | **--** | **--** | **Near IR L/D** | **--** | **LifeTech** | **1/1000** |
| **86** | **Rat serum IgG** | **--** | **--** | **--** | **--** | **10** | **Sigma** | **1/200** |
| **87** | **Fc Block CD16/32** | **2.4G2** | **Mouse** | **Rat IgG2b, κ** | **--** | **0.5** | **BD** | **1/200** |
|  |  |  |  |  |  |  |  |  |
